# Primary cilium as a sensory organelle and metabolic hub in health and diseases

**DOI:** 10.1186/s12929-026-01291-6

**Published:** 2026-10-07

**Authors:** Huan-Tzu Hou, Yu-Ying Chao, Jie-Ning Li, I-Chen Peng, Tzu-Chien Lin, Wen-Tai Chiu, Chia-Yih Wang, Shaw-Jenq Tsai

**Affiliations:** 1 Institute of Basic Medical Sciences, College of Medicine, National Cheng Kung University, Tainan, 70101 Taiwan; 2 Department of Physiology, College of Medicine, National Cheng Kung University, 1 University Road, Tainan, 70101 Taiwan; 3 Institute of Molecular Biology, Academia Sinica, Taipei, Taiwan; 4 Department of Cell Biology and Anatomy, College of Medicine, National Cheng Kung University, 1 University Road, Tainan, 70101 Taiwan; 5 Department of Life Sciences, National Cheng Kung University, Tainan City, 701 Taiwan; 6 Department of Biomedical Engineering, National Cheng Kung University, Tainan City, 701 Taiwan; 7 Medical Device Innovation Center, National Cheng Kung University, Tainan City, 701 Taiwan; 8 Department of Biomedical Sciences, College of Science, National Chung Cheng University, 168, Sec. 1, University Road, Minxiong, 621301 Chiayi Taiwan

**Keywords:** Primary cilia, Metabolic signaling, Nutrient sensing, Mechanotransduction, Glucose and lipid metabolism, Mitochondrial homeostasis, Ciliopathies, Human disease

## Abstract

Primary cilia are microtubule-based organelles protruding from the cell surface, which are conventionally understood as passive cellular antennae that detect extracellular stimuli. However, emerging evidence redefines these microtubule-based organelles as dynamic, bidirectional hubs that integrate environmental sensing with cellular metabolic adaptation. By selectively compartmentalizing receptors, ion channels, transporters, and signaling components, they function as specialized signal-processing compartments that receive, integrate, amplify, and relay information to coordinate cellular metabolism, adaptation, and cell-fate determination. Cilia-dependent signaling pathways regulate glucose and lipid metabolism, Ca^2^⁺ and second-messenger dynamics, and mitochondrial homeostasis. Conversely, environmental and metabolic changes remodel ciliary structure, molecular composition, and signaling competence. In this review, we present primary cilia as a bidirectional interface between environmental sensing and metabolic adaptation. We discuss how cilia respond to nutrient availability, mechanical forces, and oxygen tension, and how disruption of this sensory-metabolic interface contributes to metabolic, cardiovascular, reproductive, neurodegenerative, and cancer-related diseases. Defining the cellular and environmental contexts in which ciliary remodeling is protective or pathogenic will be essential for translating cilia biology into therapeutic strategies.

**Graphical Abstract:**

**Figure:**
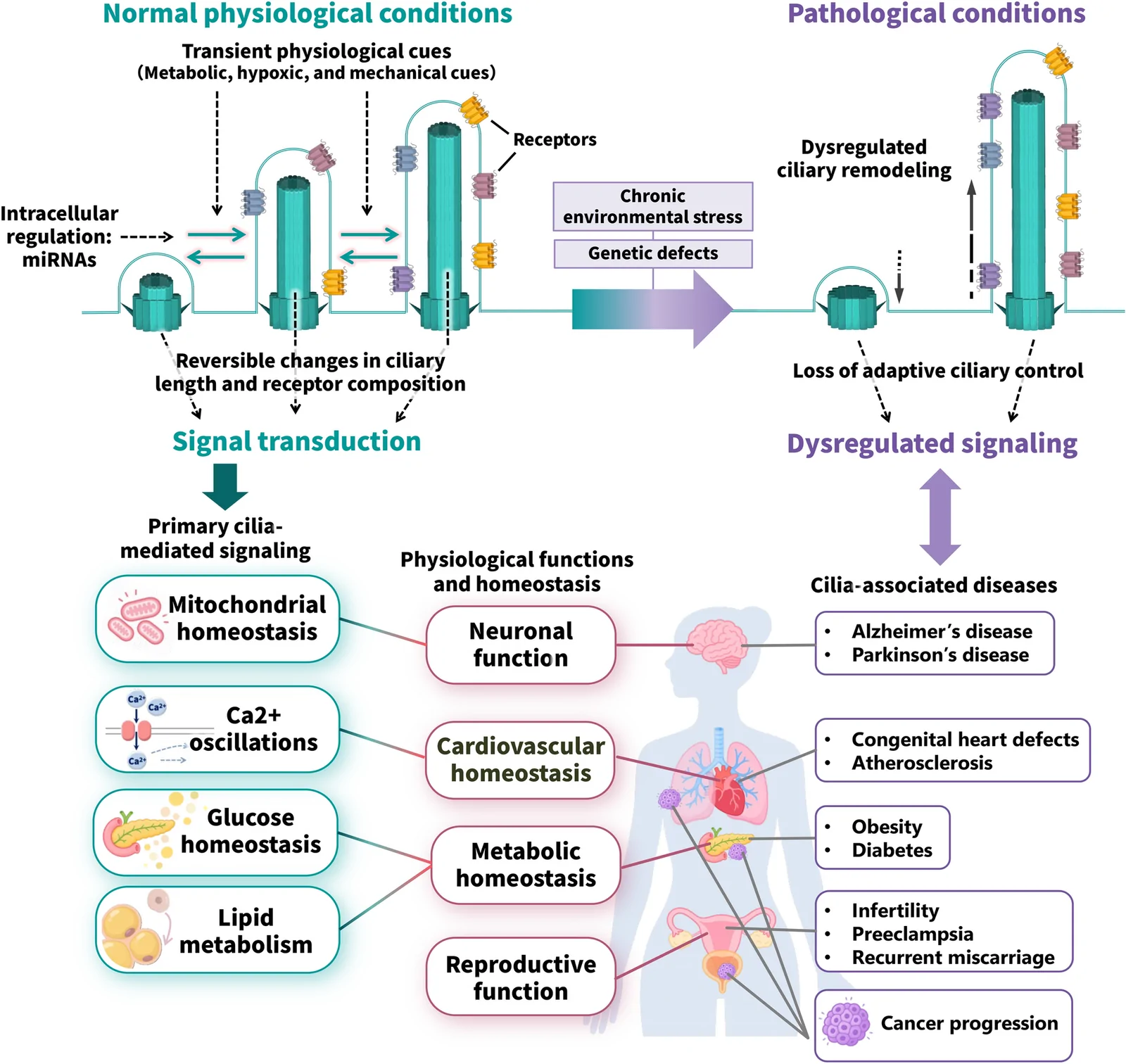

**Supplementary Information:**

The online version contains supplementary material available at https://doi.org/10.1186/s12929-026-01291-6.

## Introduction

Primary cilia are non-motile cellular antennas that extend from the cell surface, providing a specialized sensory interface through which cells perceive microenvironmental cues and convert them into cellular responses [1]. Structurally, the primary cilium is anchored by the mother centriole-derived basal body. During G0/G1 phase or cellular differentiation, the mother centriole docks to the cell membrane and nucleates the formation of a 9 + 0 axoneme, which is surrounded by a specialized ciliary membrane that is continuous with the plasma membrane. Ciliary assembly, maintenance, and signaling competence depend on tightly regulated trafficking systems. In particular, intraflagellar transport (IFT) mediates bidirectional cargo movement along the axoneme through IFT-A and IFT-B complexes associated with kinesin-2 and cytoplasmic dynein-2 motors [2–4].

The sensory capacity of primary cilia is supported by the selective enrichment of receptors, ion channels, transporters, and signaling molecules within the ciliary compartment [5, 6]. At the ciliary base, transition fibers and the transition zone act as a selective gate that regulates the entry and exit of ciliary components, thereby preserving the cilium as a distinct compartment [7]. These structural and trafficking features allow primary cilia to operate as spatially restricted signaling hubs rather than passive membrane protrusions.

Within this confined compartment, primary cilia coordinate multiple signaling pathways, including Hedgehog, G protein-coupled receptor (GPCR), Ca^2^⁺, receptor tyrosine kinase, transforming growth factor (TGF)-β/bone morphogenetic protein, and Wnt signaling [8]. Hedgehog signaling remains the most established cilia-dependent signal transduction, whereas ciliary GPCRs, ion channels, and receptor-associated pathways demonstrate the broader capacity of primary cilia to organize tissue- and stimulus-specific responses [5].

Metabolic homeostasis requires cells to continuously align nutrient uptake and utilization, energy production, biosynthesis, redox balance, and organelle quality with changing extracellular conditions. Primary cilia are well-positioned at this interface because they concentrate receptors, ion channels, and signaling machinery upstream of nutrient- and energy-responsive pathways, including liver kinase B1 (LKB1)-AMPK and mTORC1 [9, 10]. Through this spatial organization, ciliary signaling can influence endocrine output, glucose and lipid homeostasis, autophagy, and mitochondrial remodeling [10–14]. This relationship is reciprocal: nutrient availability and lipid composition can remodel cilium assembly and membrane identity. Primary cilia may therefore function as bidirectional sensory-metabolic interfaces that both respond to metabolic state and coordinate adaptive metabolic programs. Nevertheless, understanding of the cilia-metabolism axis remains incomplete and highly dependent on cell type, tissue context, and environmental conditions [15].

In this review, we use an “environmental sensing-metabolic adaptation” framework to discuss how primary cilia connect extracellular cues with intracellular metabolic states. First, we summarize how environmental inputs, including nutrient availability, mechanical forces, and oxygen tension, reshape ciliary structure and function. Second, we discuss how primary cilia participate in metabolic regulation through glucose and endocrine signaling, lipid metabolism, ciliary Ca^2^⁺ signaling, miRNA-associated regulation, and mitochondrial homeostasis. Third, we examine how disruption of this cilia-metabolism axis contributes to cardiovascular, reproductive, metabolic, cancer-related, and nervous system diseases. Finally, we highlight unresolved questions and future opportunities for targeting ciliary signaling in metabolic and disease contexts. Unlike previous reviews that mainly focus on ciliary biogenesis, classical ciliopathies, or individual signaling pathways, this review provides a comprehensive overview of the emerging role of primary cilia as sensory-metabolic interfaces that translate microenvironmental changes into adaptive or maladaptive cellular responses.

## Environmental cues and intracellular regulatory programs reshape primary cilia

Primary cilia are not static sensory organelles; rather, their assembly, length, composition, and signaling output are dynamically remodeled by changes in the extracellular environment. Nutrient availability, mechanical forces, and oxygen tension can alter ciliary structure and function, thereby shaping downstream signaling pathways involved in cellular adaptation, tissue organization, and stress responses. In this section, we discuss how major environmental cues remodel primary cilia and how these changes may influence cellular behavior under physiological and disease-relevant conditions.

### Nutrient stress and endogenous metabolic alterations remodel primary cilia

Nutrient availability and intracellular metabolic homeostasis can influence primary cilium assembly, length, membrane composition, and signaling competence [16–22]. Environmental nutrient stress, such as glucose or glutamine limitation, alters nutrient-sensitive pathways including mTORC1 and can induce context-dependent changes in ciliogenesis [16, 17]. In parallel, endogenous metabolic alterations, including lipid remodeling, phosphoinositide turnover, cholesterol distribution, and lipogenic pathway activation, can modify the ciliary membrane and trafficking machinery [18, 20–22]. Therefore, primary cilia should not be viewed as responding uniformly to individual nutrient classes. Instead, nutrient- and metabolism-dependent ciliary remodeling reflects the combined effects of extracellular nutrient availability and intracellular metabolic programs.

#### Exogenous nutrient stress and ciliary remodeling

Glucose deprivation is one of the best-characterized nutrient stresses linked to primary cilium remodeling. In cultured cells, glucose deprivation promotes primary cilium formation mainly through inactivation of mechanistic target of rapamycin complex 1 (mTORC1) rather than through autophagy-dependent mechanisms [16]. Importantly, this response is accompanied by an increased proportion of ciliated cells but shorter ciliary length, indicating that glucose deprivation differentially regulates cilium assembly and ciliary morphology [16]. Thus, glucose availability should not be interpreted as a simple switch of ciliogenesis, but rather as a cue that can separately influence whether a cilium forms and how long or structurally mature the cilium becomes.

Amino acid limitation may also influence ciliary remodeling through nutrient-sensing and stress-response pathways; however, the direct evidence that general amino acid deprivation universally induces ciliogenesis remains limited. Because amino acids are major upstream regulators of mTORC1, amino acid deprivation may create a metabolic state permissive for ciliary remodeling [23, 24]; however, this effect is likely context-dependent rather than uniform across cell types or amino acid species. Among individual amino acids, glutamine provides the clearest example of nutrient-specific ciliary regulation. In pancreatic ductal adenocarcinoma, glutamine deprivation induces melanophilin, which accumulates at the basal body and promotes primary cilium regrowth [17]. These reassembled cilia enhance phospholipase C (PLC)-γ1 expression and ciliary localization, forming a feedforward signaling circuit that promotes metastatic phenotypes. Thus, glutamine deprivation illustrates how a specific exogenous nutrient stress can remodel primary cilia and generate disease-relevant signaling outputs.

#### Endogenous metabolic alterations and lipid-dependent ciliary regulation

In addition to exogenous nutrient availability, endogenous metabolic alterations can regulate primary cilia by remodeling ciliary membrane identity, trafficking, and signaling competence. Phosphoinositide homeostasis contributes to basal body maturation, axoneme initiation, ciliary membrane identity, and ciliary protein trafficking. Local phosphatidylinositol-4-phosphate remodeling at the centrosome promotes tau tubulin kinase 2 recruitment to the mother centriole, triggering removal of centriolar coiled-coil protein 110 (CP110), which normally blocks axoneme extension, and thereby initiating primary cilium formation [18]. Cholesterol is another important regulator of ciliary signaling competence, particularly in the Hedgehog pathway, where cholesterol accessibility within the ciliary membrane modulates Smoothened activity [19]. Cholesterol distribution can also affect ciliary membrane organization and ciliogenesis through changes in membrane properties [20].

Endogenous lipogenic programs can also regulate ciliogenesis. Activation of sterol regulatory element-binding protein 1 (SREBP1)-driven lipogenesis and enhanced fatty acid synthesis suppress primary cilium formation [21, 22], in part by altering vesicular trafficking pathways. Because these mechanisms arise from intracellular metabolic reprogramming rather than extracellular nutrient availability, we now discuss them as endogenous metabolic alterations rather than as examples of exogenous nutrient stress. Together, these findings indicate that primary cilia are regulated by both extracellular nutrient stress and intracellular metabolic programs, with outcomes that depend on cell type, metabolic state, and signaling context.

### Mechanical forces remodel primary cilium structure and signaling

Cells in different tissues are continuously exposed to mechanical inputs, including fluid shear stress, compression, matrix stiffness, and hydrostatic pressure. Primary cilia can convert these physical cues into intracellular signaling responses, thereby linking mechanical information to tissue homeostasis. However, mechanical stimulation does not uniformly induce ciliogenesis. Depending on the tissue context, force pattern, and duration, mechanical cues may bend, elongate, shorten, or promote resorption of primary cilia.

#### Shear stress

Fluid shear stress is one of the best-characterized mechanical cues sensed by primary cilia in fluid-filled tissues such as renal tubules and specific vascular regions [25, 26]. In renal epithelial cells, early studies proposed that urinary flow bends the apical primary cilium and activates Ca^2^⁺ signaling through the polycystin-1–polycystin-2 (PC1–PC2; encoded by *PKD1* and *PKD2*, respectively) complex [25]. Disruption of these proteins impairs flow-induced Ca^2+^ responses, supporting their role in cilia-mediated mechanotransduction. Cilia-mediated flow sensing also regulates shear stress-dependent apical endocytosis in renal proximal tubule cells, suggesting a role in tubular reabsorptive function [27]. In vascular endothelial cells, primary cilia are particularly sensitive to the local hemodynamic environment. They are commonly retained under static or disturbed-flow conditions, whereas sustained laminar shear stress can promote ciliary shortening or disassembly [28]. These structural changes alter the capacity of endothelial cells to generate cilia-dependent signaling outputs, including nitric oxide production, and may thereby influence endothelial homeostasis [26]. Thus, endothelial primary cilia appear to act as flow-responsive organelles whose presence and signaling output depend on the local hemodynamic environment.

#### Mechanical loading and matrix stiffness

Mechanical loading can alter primary ciliary morphology and signaling competence in mechanically active tissues such as bone and cartilage. In bone cells, primary cilia deflect in response to dynamic fluid flow and are required for osteogenic and bone-resorptive responses [29]. In osteoblasts, cilia mediate oscillatory flow-induced Ca^2+^ deposition, linking force-dependent ciliary activity to bone formation [30]. In chondrocytes, primary cilia are required for compression-induced ATP release, which is associated with extracellular matrix regulation [31]. These findings indicate that primary cilia help translate mechanical loading into tissue-specific biochemical responses. Primary cilia also respond to the mechanical properties of the extracellular matrix. Changes in substrate stiffness regulate ciliary number, length, and centriole positioning, suggesting that the cilium-centrosome axis adapts to different extracellular matrix environments [32]. Stiff extracellular matrices can suppress primary ciliogenesis by enhancing actomyosin tension-dependent nuclear deformation and upregulation of keratin-associated protein [33]. Collectively, these findings show that mechanical cues dynamically remodel primary ciliary structure and signaling.

### Oxygen tension modulates ciliary structure and signaling

Oxygen availability is an important environmental cue that regulates cellular metabolism and stress adaptation. Under hypoxic conditions, cells activate hypoxia-inducible factor (HIF)-dependent transcriptional programs to adjust energy production, redox balance, and survival responses [34]. Emerging evidence suggests that primary cilia are connected to this oxygen-responsive signaling network, although their role in hypoxia sensing appears to be context-dependent. One mechanism linking oxygen-responsive signaling to primary cilia involves the von Hippel-Lindau protein (pVHL), an E3 ubiquitin ligase that regulates HIF-α degradation. pVHL localizes to the primary cilium and basal body and contributes to microtubule stability and cilium maintenance [35]. Consistently, loss of pVHL impairs ciliogenesis in renal-derived cells, whereas restoration of functional pVHL can rescue cilium formation [36]. In addition, HIF-2α can interact with IFT88 under hypoxic conditions to promote ciliogenesis and influence MEK/ERK signaling through a primary cilium-associated mechanism [37]. Together, these findings suggest that primary cilia may function as context-dependent platforms that connect oxygen-responsive signaling to localized ciliary regulation, rather than acting as universal hypoxia sensors.

### Intracellular miRNA programs regulate ciliogenesis

Unlike nutrient availability, mechanical force, and oxygen tension, miRNAs are not extracellular environmental cues. Instead, miRNAs and the RNA interference machinery function as intracellular post-transcriptional regulators that shape primary cilium assembly, length, and structural specialization by controlling centrosomal proteins, cytoskeletal organization, and gene expression. In human astrocytes, the RNA-silencing proteins GW182 and Argonaute 2 localize near the centrosome and basal body, and depletion of these RNAi effectors or key miRNA-processing factors impairs axoneme formation and elongation, suggesting that the miRNA biogenesis and effector machinery is required for primary cilium maturation [38]. miR-129-3p promotes primary ciliogenesis by targeting CP110 and modulating actin dynamics, linking post-transcriptional regulation to centrosome remodeling [39]. Beyond cilium initiation, miRNAs can also regulate ciliary architecture after assembly. miR-669a-5p promotes primary cilium elongation by suppressing ciliary disassembly-associated pathways [40]. In specialized sensory tissues, miR-182 and miR-183 are critical for maintaining cone photoreceptor outer segments, which are highly specialized primary cilia required for visual function [41]. Together, these studies establish miRNAs as intracellular regulators of cilium formation, length, stability, and tissue-specific architecture rather than as environmental inputs. More broadly, primary cilia are shaped by two conceptually distinct classes of signals: extracellular environmental cues and intracellular regulatory programs. This distinction provides a framework for interpreting context-dependent ciliary plasticity and its downstream effects on cellular adaptation.

## Primary cilia as metabolic signaling hubs

After sensing environmental cues, primary cilia can further shape cellular metabolism by organizing signaling events into localized and stimulus-specific outputs. In this context, the primary cilium acts not only as an upstream sensor but also as a compartmentalized signaling platform that helps translate extracellular information into metabolic responses. Understanding how primary cilia coordinate metabolic signaling, therefore, provides a framework for interpreting both physiological adaptation and disease progression.

### Ciliary signaling in pancreatic islet function and glucose homeostasis

**Fig. 1:**
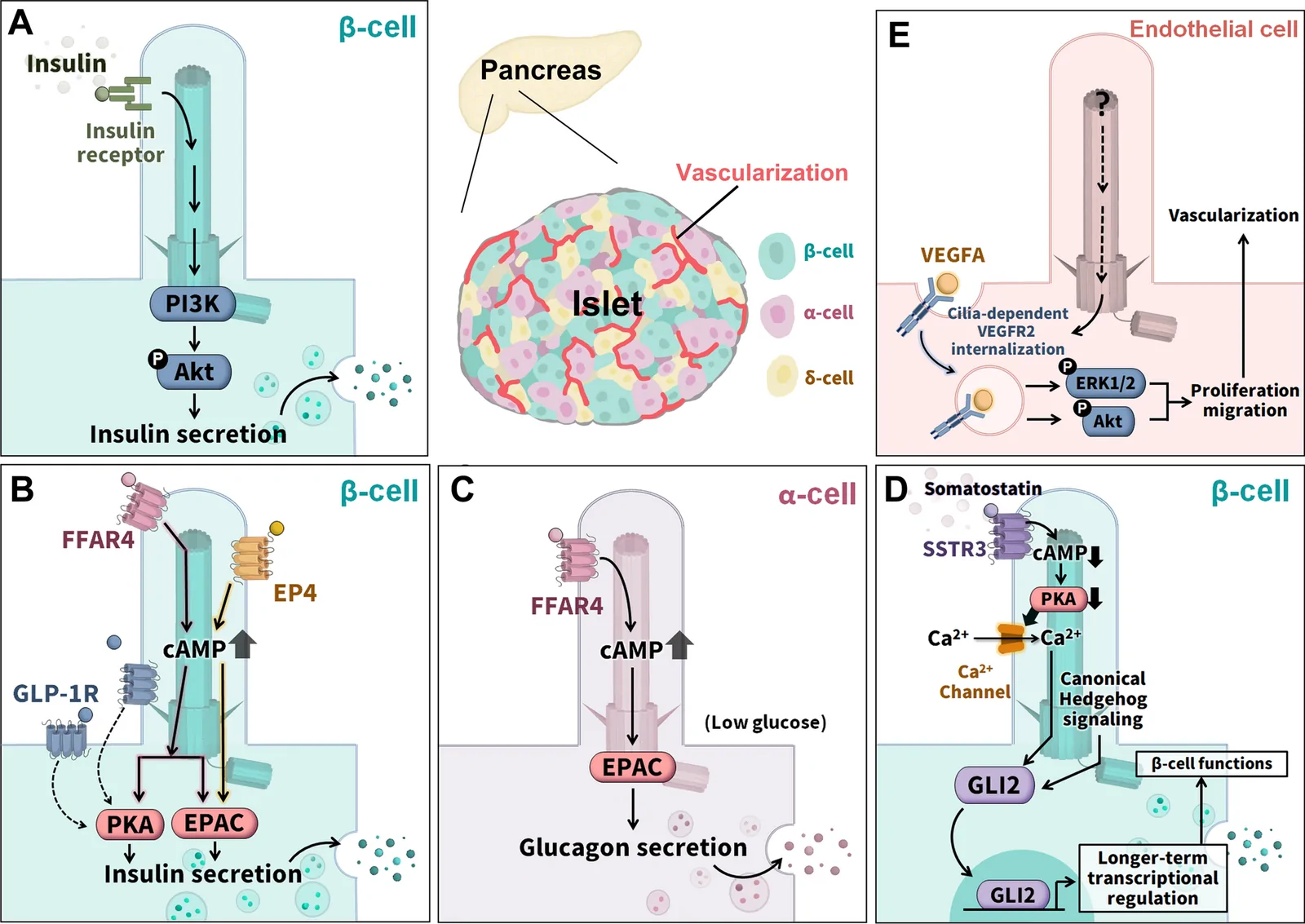
Ciliary signaling in pancreatic islet function and glucose homeostasis. The central schematic depicts a vascularized islet containing α-, β-, and δ-cells. **A** In β-cells, insulin recruits insulin receptor isoform A to the primary cilium and activates PI3K/Akt signaling to support insulin secretion. **B** In β-cells, ciliary FFAR4 signals through PKA and EPAC, whereas EP4 primarily signals through EPAC to enhance insulin secretion. Ciliary and nonciliary GLP-1R pools also contribute to cAMP-dependent insulin secretion. **C** In α-cells, ciliary FFAR4 activates cAMP/EPAC signaling to promote glucagon secretion under low-glucose conditions. **D** Somatostatin activates ciliary SSTR3, reducing cAMP/PKA signaling and inducing ciliary Ca^2^⁺ signaling, which triggers GLI2 nuclear translocation. This connects ciliary GPCR signaling to the Hedgehog/GLI axis, which supports the long-term maintenance of β-cell function. **E** Endothelial primary cilia facilitate VEGFR2 internalization and ERK1/2–Akt signaling, supporting endothelial proliferation, migration, and islet vascularization. Solid arrows indicate experimentally supported signaling relationships, whereas dashed arrows indicate incompletely defined connections

Primary cilia are present on pancreatic islet cells, where they contribute to glucose homeostasis by organizing endocrine and paracrine signaling [42]. Rather than functioning universally as direct glucose sensors, islet primary cilia compartmentalize receptors and downstream signaling molecules that regulate hormone secretion (Fig. 1). Disruption of β-cell ciliogenesis or ciliary signaling impairs insulin secretion and glucose tolerance [12, 43]. In β-cells, insulin stimulation recruits insulin receptor isoform A to the primary cilium, while disruption of ciliary or basal body integrity attenuates downstream phosphoinositide 3-kinase (PI3K)/Akt signaling (Fig. 1A) [12]. Bardet–Biedl syndrome (BBS) proteins form the BBSome, a ciliary trafficking complex [15]. In *Bbs4*-deficient mice, ciliary dysfunction is associated with delayed glucose clearance and impaired β-cell insulin secretion [12]. Similarly, β-cell-specific *Ift88* deletion disrupts endosomal processing of Ephrin type-A (EphA) receptors, resulting in abnormal EphA3 signaling and impaired glucose-stimulated insulin secretion [44]. These findings indicate that β-cell primary cilia support insulin secretion by coordinating receptor trafficking, intracellular signaling, and secretory responses.

Cilia-localized GPCRs constitute an additional regulatory axis in islet hormone secretion. Free fatty acid receptor 4 (FFAR4) is enriched in the primary cilia of mouse and human pancreatic α- and β-cells, whereas prostaglandin E₂ receptor 4 (EP4) is predominantly localized to β-cell cilia and is minimally present in α-cell cilia [42]. In β-cells, activation of either receptor enhances glucose-stimulated insulin secretion via cyclic adenosine monophosphate (cAMP)-dependent signaling pathways. FFAR4 signals through both protein kinase A (PKA) and exchange protein directly activated by cAMP (EPAC), whereas EP4 signaling is primarily mediated by EPAC (Fig. 1B). In α-cells, FFAR4 activation elevates ciliary cAMP levels and augments glucagon secretion via EPAC under low-glucose conditions (Fig. 1C). Knockdown of tubby-like protein 3 (TULP3) disrupts ciliary localization of these receptors and diminishes their functional responses, implicating the physiological relevance of these ciliary GPCR pools [42].

Somatostatin receptor 3 (SSTR3) is enriched in primary cilia of mouse and human β-cells [45]. Somatostatin released from neighboring δ-cells activates ciliary SSTR3, lowering ciliary cAMP and inducing cilia-restricted Ca^2^⁺ signaling that promotes GLI2 nuclear translocation. This connects ciliary GPCR signaling to a key downstream effector of the Hedgehog pathway (Fig. 1D) [45]. Tight control of GLI/Hedgehog activity is important for β-cell identity and function: in mouse models, constitutive GLI2 activation reduced insulin expression and glucose-stimulated insulin secretion, leading to glucose intolerance, while concomitant cilia loss further exacerbated these defects [45, 46]. These findings suggest that ciliary control of GLI/Hedgehog activity links local GPCR signaling to the maintenance of β-cell functions.

Glucagon-like peptide-1 receptor (GLP-1R) classically signals from the plasma membrane and endosomes through cAMP and Ca^2^⁺ pathways to potentiate glucose-stimulated insulin secretion [47]. Co-activation of GLP-1R and FFAR4 further enhances insulin secretion, suggesting cooperation between their downstream pathways (Fig. 1B), although the GLP-1R compartment involved was not determined [42]. Recent evidence identified endogenous GLP-1R on mouse β-cell primary cilia [48]. Loss of β-cell cilia attenuated GLP-1R agonist-induced cAMP and Ca^2^⁺ signaling and insulin secretion in mouse islets, while IFT88 knockdown similarly impaired GLP-1R agonist-potentiated insulin secretion in human islets. TULP3 knockdown selectively reduced ciliary GLP-1R localization and blunted cAMP signaling and insulin secretion induced by liraglutide, a GLP-1R agonist. Ciliary PKA inhibition also reduced this secretory response, supporting a functional ciliary GLP-1R–cAMP–PKA axis [48]. Collectively, these cilia-localized GPCRs integrate paracrine, endocrine, and nutrient-derived signals to regulate insulin and glucagon secretion across islet endocrine cell types. Endothelial primary cilia may further support islet glucose homeostasis by maintaining vascular function. In *Bbs4*-deficient mice, reduced intra-islet capillary density and capillary fenestration impair vascular permeability and glucose delivery to β-cells. Mechanistically, depletion of BBS4 or IFT88 disrupts vascular endothelial growth factor A (VEGF-A) uptake and ligand-induced vascular endothelial growth factor receptor 2 (VEGFR2) internalization. Because VEGFR2 signals primarily from endosomal compartments, impaired internalization reduces VEGFR2 phosphorylation and downstream ERK1/2 and Akt activation, thereby attenuating endothelial proliferation and migration (Fig. 1E) [49]. Thus, ciliary regulation of glucose homeostasis encompasses both endocrine signaling and vascular support within the islet microenvironment.

### Ciliary regulation of lipid metabolism

**Fig. 2:**
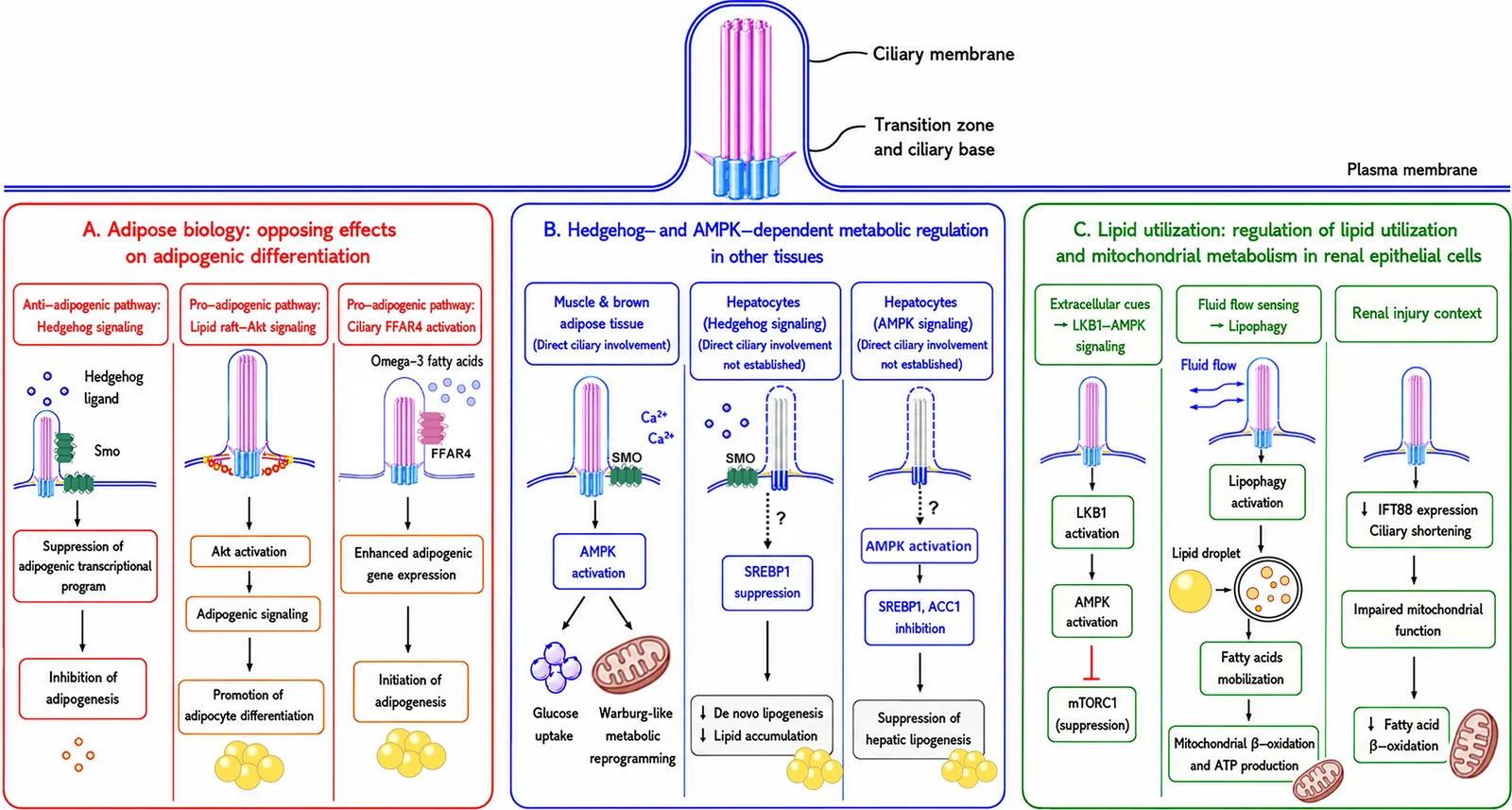
Context-dependent regulation of lipid metabolism by primary cilia and associated signaling pathways. **A** In adipose biology, distinct ciliary mechanisms exert opposing effects on adipogenic differentiation. Primary cilium-dependent Hedgehog signaling suppresses the adipogenic transcriptional program, whereas lipid-raft–Akt signaling at the ciliary base and activation of ciliary FFAR4 by omega-3 fatty acids promote adipocyte differentiation. **B** In muscle and brown adipose tissue, cilium-dependent Smoothened–Ca^2^⁺ signaling activates AMPK, promoting glucose uptake and Warburg-like metabolic reprogramming. Hedgehog and AMPK signaling suppress SREBP1-dependent lipogenesis in hepatocytes; however, direct involvement of primary cilia in these hepatic effects has not been established. **C** In renal epithelial cells, primary cilia couple extracellular cues to LKB1–AMPK signaling and mTORC1 suppression and sense fluid flow to promote lipophagy, fatty acid mobilization, mitochondrial β-oxidation, and ATP production. In renal injury, reduced IFT88 expression and ciliary shortening are associated with impaired mitochondrial function and fatty acid oxidation. Collectively, these findings illustrate tissue- and context-dependent relationships between primary cilia, adipogenesis, lipogenesis, and lipid utilization. Solid arrows indicate experimentally demonstrated relationships within the cited systems; dashed arrows indicate predicted or inferred links

Primary cilia influence lipid metabolism most clearly in adipose biology, where distinct ciliary mechanisms exert opposing effects on adipogenic differentiation [50] (Fig. 2). Primary cilium-dependent Hedgehog signaling suppresses the adipogenic transcriptional program, thereby inhibiting adipogenesis [51]. In contrast, lipid-raft organization at the ciliary base supports Akt-dependent adipogenic signaling, thereby promoting adipocyte differentiation [52], whereas activation of ciliary FFAR4 by omega-3 fatty acids enhances adipogenic gene expression and initiates adipogenesis [53]. Together, these findings demonstrate that primary cilia integrate distinct anti- and pro-adipogenic signaling pathways rather than functioning through a single linear mechanism.

Beyond adipogenesis, ciliary signaling may also be linked to metabolic regulation through Hedgehog- and AMPK-dependent pathways in other tissues (Fig. 2), although the evidence for direct ciliary involvement varies among cellular contexts. In muscle and brown adipose tissue, cilium-dependent Smoothened-Ca^2^⁺ signaling activates AMPK, promoting glucose uptake and Warburg-like metabolic reprogramming [54]. In hepatocytes, Hedgehog signaling suppresses SREBP1-dependent de novo lipogenesis and lipid accumulation [55], whereas AMPK suppresses hepatic lipogenesis by inhibiting SREBP1 and acetyl-CoA carboxylase 1 (ACC1) [56]; however, whether these metabolic effects involve primary cilia remains to be established. Together, these findings suggest a potential connection between ciliary signaling and Hedgehog- and AMPK-associated regulation of lipogenesis. Importantly, the supporting evidence derives from distinct tissue contexts and should not be generalized across tissues.

Primary cilia also contribute to the regulation of lipid utilization (Fig. 2). In renal epithelial cells, primary cilia couple extracellular cues to LKB1-AMPK signaling and suppress mTORC1 activity [57]. Primary cilia also sense fluid flow to promote lipophagy, thereby mobilizing fatty acids from lipid droplets for mitochondrial β-oxidation and ATP production [10]. In a distinct renal injury context, decreased IFT88 expression and ciliary shortening are associated with impaired mitochondrial function and fatty acid oxidation in renal tubular epithelial cells [58]. Collectively, these studies support a role for primary cilia in regulating lipid utilization and mitochondrial metabolism in renal epithelial cells. Overall, these findings highlight context-dependent links between primary cilia and adipogenic differentiation, lipogenesis, and lipid utilization, with the underlying mechanisms and strength of evidence varying across tissues.

### Ciliary regulation of Ca^2^⁺ and second-messenger signaling

**Fig. 3:**
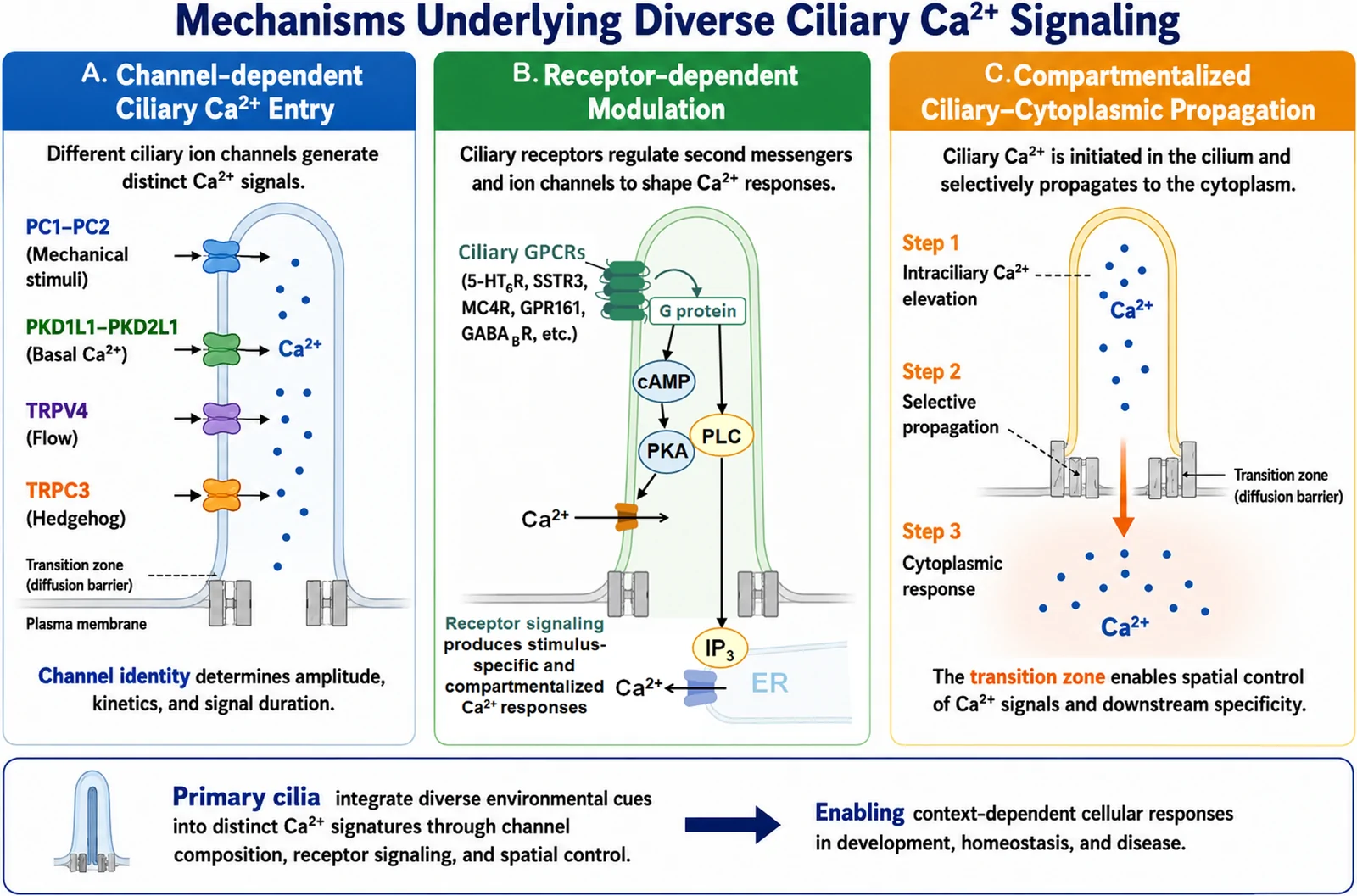
Mechanisms underlying diverse ciliary Ca^2^⁺ signaling. Primary cilia function as spatially restricted signaling compartments that convert extracellular cues into localized Ca^2^⁺ and second-messenger responses. **A** Channel-dependent ciliary Ca^2^⁺ signaling. Ciliary ion channels, including PC1-PC2, polycystin-1-like 1-polycystin-2-like 1, TRPV4, and TRPC3, contribute to stimulus- and cell type-specific Ca^2^⁺ signals within the ciliary compartment. **B** GPCR-dependent second-messenger signaling. Cilia-localized GPCRs regulate local cAMP and PLC-associated pathways, thereby modulating PKA activity, Ca^2^⁺-permeable channels, and intracellular Ca^2^⁺ release. **C** Compartmentalized signaling outputs. The transition zone restricts exchange between the cilium and cytoplasm, allowing ciliary Ca^2^⁺ and cAMP signals to remain locally restricted or to engage broader cytoplasmic signaling networks depending on cellular context. Together, these mechanisms support the role of primary cilia as compartmentalized signaling hubs that translate environmental cues into context-dependent cellular responses

Primary cilia generate localized signaling outputs through compartmentalized Ca^2^⁺ and second-messenger dynamics. Rather than functioning as uniform Ca^2^⁺ entry structures, primary cilia produce stimulus- and cell type-specific signals shaped by channel composition, receptor localization, and diffusion barriers (Fig. 3). This compartmentalization allows cilia to convert extracellular inputs into spatially restricted signaling events. Several ciliary ion channels contribute to Ca^2^⁺ signaling within the ciliary compartment. The PC1–PC2 complex is one of the most studied ciliary channel systems and has been implicated in renal epithelial and endothelial responses to mechanical bending or fluid shear stress [25, 26, 59]. However, physiological mechanical stimulation does not always generate detectable ciliary Ca^2^⁺ responses, indicating that ciliary Ca^2^⁺ signaling is highly cell type- and context-dependent [60, 61]. The polycystin-1-like 1–polycystin-2-like 1 (PKD1L1–PKD2L1) complex is also enriched in primary cilia and supports intraciliary Ca^2^⁺ currents, contributing to the distinct basal Ca^2^⁺ concentration within the cilioplasm [62]. Other transient receptor potential (TRP) channels, including the vanilloid subtype 4 (TRPV4) and canonical subtype 3 (TRPC3), further diversify ciliary Ca^2^⁺ responses across different tissues and signaling contexts [63, 64]. Thus, ciliary Ca^2^⁺ signaling is determined by channel identity, stimulus, and cellular context rather than by a single universal mechanism.

Ciliary receptors provide an additional layer of control over Ca^2^⁺ and second-messenger signaling. GPCRs enriched in the ciliary membrane can regulate compartmentalized cAMP signaling and PLC-associated pathways, thereby modulating local protein kinase A activity and ciliary ion-channel function. Importantly, PLC/IP3-dependent Ca^2^⁺ release should be distinguished from intraciliary Ca^2^⁺ signaling. Because the endoplasmic reticulum is not present within the ciliary compartment, IP3 receptor-mediated Ca^2^⁺ release occurs from intracellular Ca^2^⁺ stores outside the cilium and contributes to broader cytosolic Ca^2^⁺ responses. The transition zone contributes to the spatial compartmentalization of ciliary signaling while allowing cilium-initiated pathways to influence broader cytoplasmic signaling networks [7]. For example, in neural cilia, sonic hedgehog signaling has been associated with TRPC3-dependent ciliary Ca^2^⁺ signaling together with intracellular Ca^2^⁺ release from extraciliary stores [64]. Thus, ciliary Ca^2^⁺ and second-messenger signaling should be viewed as a flexible, compartmentalized system in which ciliary ion channels generate localized intraciliary signals, whereas receptor-initiated signaling can also engage extraciliary Ca^2^⁺-release pathways and broader cytoplasmic signaling networks depending on cellular context.

### Ciliary trafficking and EV-associated miRNA signaling

Direct evidence that primary cilia selectively package or secrete miRNAs remains limited. Current studies confirm that both mammalian and invertebrate primary cilia release extracellular vesicles (EVs) carrying specific protein cargo, such as GPCRs and polycystin-related proteins, respectively [65–67]. These studies establish the release of cilia-associated EVs containing defined protein cargo, but whether the miRNA loading into such vesicles remains unclear. Although mammalian EVs generally carry diverse bioactive cargo, including RNAs, proteins, and lipids [65, 68], the specific characterization of miRNA cargo within verified ciliary-derived vesicles has yet to be demonstrated.

**Fig. 4:**
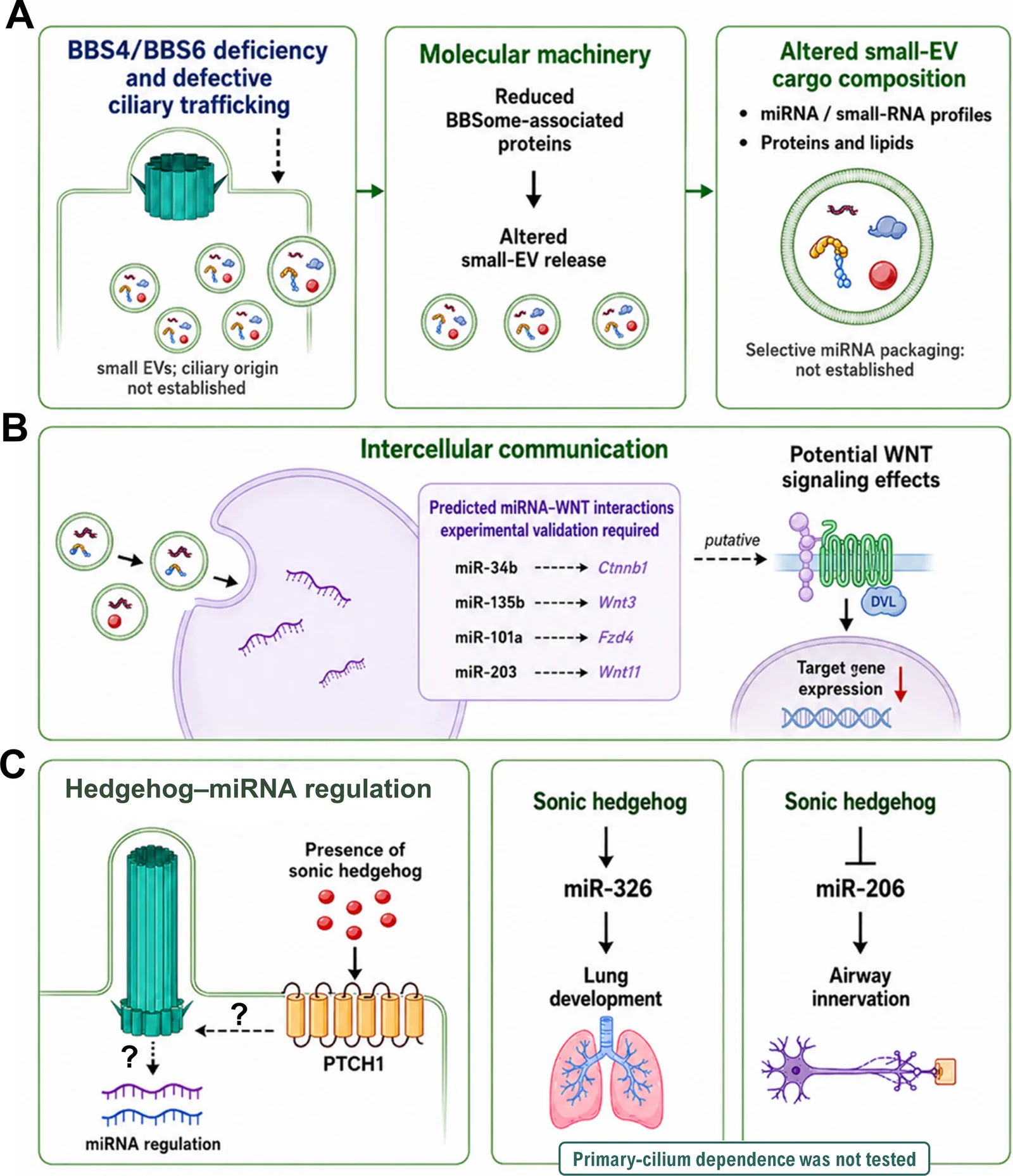
Evidence boundaries in cilia–EV–miRNA signaling. **A** In immortalized murine kidney medullary cells, *Bbs4* or *Bbs6* loss disrupts BBSome-associated ciliary trafficking, increases release of multivesicular-body-associated small EVs, and changes EV miRNA, protein, and lipid cargo. Because these vesicles were collected from whole-cell culture supernatants, their direct origin from the ciliary membrane was not established. **B** Differentially loaded miRNAs include predicted WNT-related interactions such as miR-34b–*Ctnnb1*, miR-135b–*Wnt3*, miR-101a–*Fzd4*, and miR-203–*Wnt11*; these target assignments require experimental validation, and the reported WNT reporter effect was not shown to be miRNA-dependent. **C** Hedgehog–miRNA regulation. In embryonic lung models, Sonic hedgehog induces miR-326 in association with lung development and suppresses miR-206 in a pathway that promotes airway innervation. Because neither study tested the requirement for primary cilia, these findings support Hedgehog–miRNA regulation but do not establish a primary-cilium-dependent mechanism. Solid arrows indicate experimentally demonstrated relationships within the cited systems; dashed arrows indicate predicted or inferred links. Selective packaging of defined miRNAs into bona fide mammalian cilia-derived EVs remains unproven

Evidence linking ciliary dysfunction to EV-associated miRNA remodeling comes from immortalized murine kidney medullary cells lacking Bbs4 or Bbs6 [69]. Loss of Bbs4 or Bbs6 disrupts BBSome-associated ciliary trafficking, increases the release of multivesicular-body-associated small EVs, and alters vesicular miRNA, protein, and lipid cargo. Importantly, these small EVs were collected from whole-cell culture supernatants; their direct derivation from the ciliary membrane was not demonstrated. Twenty-four miRNAs were differentially loaded, and more than half had bioinformatically predicted WNT-related targets, including miR-34b–Ctnnb1, miR-135b–Wnt3, miR-101a–Fzd4, and miR-203–Wnt11. These predicted miRNA–target pairs require direct experimental validation. The vesicles altered WNT reporter responses in recipient cells; however, perturbing miRNA biogenesis did not change this reporter effect, suggesting that EV proteins rather than miRNAs may have been the principal drivers in that assay. Thus, this study supports ciliary-trafficking-dependent regulation of small-EV abundance and composition, not selective miRNA packaging or secretion by primary cilia (Fig. 4) [69].

EV-carried miRNAs can also act upstream of ciliogenesis. Astrocyte-derived EV miR-106b-5p targets CEP97 and promotes morphine-induced primary ciliogenesis in astrocytes [70]. These EVs were not identified as cilia-derived. Separately, studies in embryonic lung models reported Hedgehog–miRNA crosstalk [71, 72]. Sonic hedgehog induced miR-326, which feeds back on the pathway by targeting Smoothened and GLI2, and suppressed miR-206 in a Shh/miR-206/BDNF cascade controlling airway innervation [71, 72]. Neither study used cilium-deficient models, ciliary-trafficking perturbations, or compartment-specific assays; therefore, these data support Hedgehog–miRNA regulation but do not establish a primary-cilium-dependent mechanism. Heterozygous Pkd1 loss was associated with altered renal miRNA–gene networks, including predicted interactions involving miR-9-5p and miR-17-5p and genes related to lipid metabolism and tubular function [73]. These associations were primarily bioinformatic and require experimental validation. Overall, current evidence supports three distinct relationships: miRNAs can regulate ciliogenesis; ciliary-trafficking defects can alter the abundance and composition of cell-derived small EVs; and Hedgehog signaling can interact with miRNA programs. Direct selective loading of defined miRNAs into bona fide mammalian cilia-derived ectosomes remains unproven.

### Ciliary regulation of mitochondrial and metabolic homeostasis

Mitochondria are central regulators of ATP production, redox balance, and metabolic adaptation. Emerging evidence suggests a functional connection between primary cilia and mitochondrial metabolism; however, the underlying mechanisms appear to be highly cell type- and context-dependent. The best-characterized evidence comes from mechanically responsive renal epithelial cells, in which cilia-dependent sensing of fluid flow is coupled to autophagic and metabolic adaptation.

In renal epithelial cells, continuous laminar flow activates autophagy, whereas disruption of ciliary genes such as IFT88 or KIF3A attenuates this response, supporting a role for primary cilia in flow-induced autophagic adaptation [14]. Prolonged shear stress also promotes primary cilium-dependent mitochondrial biogenesis, accompanied by increased mitochondrial mass, mitochondrial DNA content, oxidative phosphorylation, and ATP production. These responses have been associated with AMPK–peroxisome proliferator-activated receptor gamma coactivator 1-alpha signaling and transcription factor EB-dependent autophagic and lysosomal programs [10]. Importantly, current evidence does not establish autophagy or mitophagy as a common upstream mechanism controlling all aspects of mitochondrial remodeling. Rather, autophagy, mitochondrial biogenesis, and other mitochondrial responses should be considered potentially interconnected but distinct adaptive processes downstream of cilia-dependent mechanosensing. Primary cilia may also influence mitochondrial dynamics in specific cellular contexts. In HEK293, U2OS, and HeLa cell models, cilia-associated proteins have been implicated in the regulation of mitochondrial dynamics [74]. Specifically, overexpression of the ciliary protein NIMA-related kinase 4 (NEK4) promotes ERK phosphorylation, followed by dynamin-related protein 1 (DRP1) activation and mitochondrial fission, providing evidence for a potential link between ciliary components and mitochondrial dynamics. However, this evidence is derived from specific cell-line models, and whether cilia regulate mitochondrial dynamics through a similar mechanism in normal tissues or other physiological contexts remains to be established.

Mechanical stimulation can additionally activate LKB1 near the ciliary basal-body compartment, leading to AMPK phosphorylation [9]. AMPK subsequently suppresses mTORC1 signaling and promotes catabolic and metabolic adaptation. Collectively, these studies support a model in which primary cilia can couple extracellular mechanical cues to autophagic and mitochondrial metabolic responses. However, the specific downstream processes and their causal relationships vary among cellular contexts and remain incompletely defined.

**Fig. 5:**
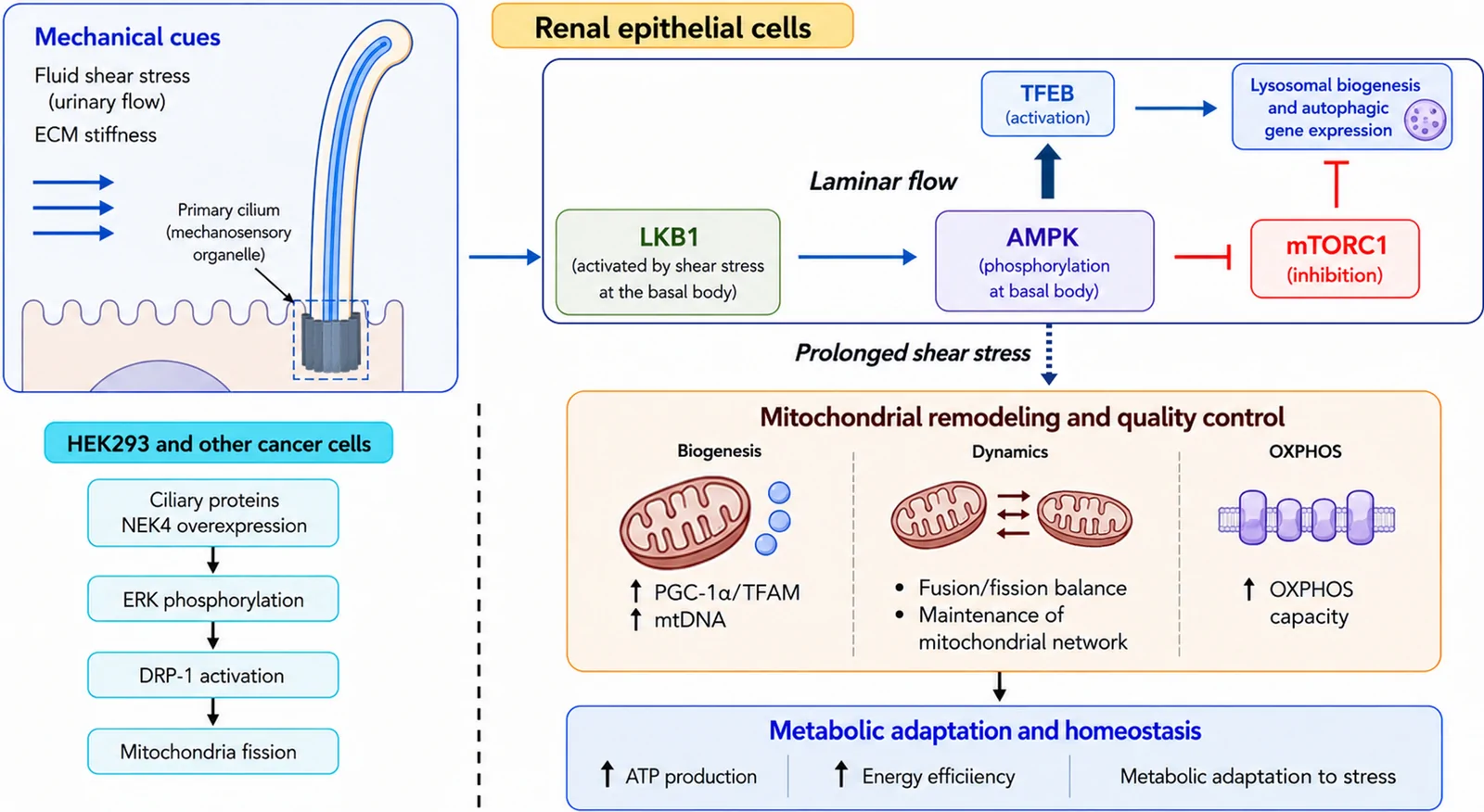
Primary cilia-associated signaling and mitochondrial homeostasis in different experimental contexts. In renal epithelial cells, mechanical stimulation activates cilium-associated LKB1–AMPK signaling, which modulates mTORC1, TFEB, autophagy, and mitochondrial homeostasis. Mitochondrial biogenesis, dynamics, and OXPHOS are presented as interconnected processes rather than sequential events downstream of mitophagy. In HEK293 and other cancer cells, cilia-associated proteins have also been linked to ERK–DRP1-dependent mitochondrial fission, although its relevance to normal tissues remains to be established

It should be noted that the individual components of this pathway have not all been demonstrated sequentially within a single experimental system. In renal epithelial models, fluid flow has been shown to induce primary cilium-dependent autophagy, whereas prolonged shear stress has been linked to cilium-dependent mitochondrial biogenesis and enhanced oxidative metabolism. Cilia-associated LKB1–AMPK signaling and mTORC1 regulation have been established in related mechanometabolic studies. Thus, the pathway illustrated in Fig. 5 represents an integrated model assembled from complementary studies rather than a fully validated linear signaling cascade.

## Cilia-metabolism axis in disease

Disruption of cilia-dependent metabolic regulation has been increasingly implicated in a broad spectrum of human diseases. Given the central role of primary cilia in integrating extracellular cues with intracellular signaling and metabolic adaptation, defects in ciliary structure, trafficking, or signaling can alter tissue homeostasis, stress responses, and disease progression. In this section, we discuss how dysregulation of the cilia–metabolism axis contributes to cardiovascular, reproductive, metabolic, cancer-related, and nervous system diseases.

### Cardiovascular system

The cardiovascular system is continuously exposed to dynamic changes in blood flow, oxygen availability, and metabolic demand. Primary cilia contribute to cardiovascular homeostasis by integrating mechanical, hypoxic, and biochemical cues that regulate endothelial function, vascular remodeling, and cardiac development. Disruption of these cilia-dependent sensing mechanisms has been linked to congenital heart defects, atherosclerosis, and defective angiogenic responses.

#### Congenital heart defects

During heart development, defects in cilia formation or cilia-dependent signaling are associated with congenital heart defects, particularly atrioventricular septal defects, in which the atrioventricular septum and valves fail to develop properly [75, 76]. Several cilia-associated genes, including the nephronophthisis-associated genes *INVS*/*NPHP2*, *NPHP3*, and *NPHP4*, have also been linked to laterality defects and structural heart malformations [77–79]. Mechanistically, primary cilia contribute to cardiac development through both biochemical and mechanical signaling. In murine cardiomyocytes, knockdown of *Ift88* or *Ift20* suppresses Hedgehog signaling, reduces cardiac differentiation markers, and impairs the formation of beating cardiomyocyte clusters [80]. Similarly, disruption of ciliary function in second heart field progenitors impairs Hedgehog signaling and atrioventricular septum formation [75]. In zebrafish, endocardial primary cilia sense low-magnitude shear stress generated by cardiac contraction and promote ventricular trabeculation and maturation [81]. Together, these findings suggest that primary cilia integrate biochemical and mechanical cues to guide cardiac morphogenesis.

#### Atherosclerosis and endothelial dysfunction

Endothelial primary cilia act as luminal sensory organelles that detect blood flow-derived shear stress and contribute to vascular homeostasis [82]. The ciliary PC1/PC2 complex has been implicated in endothelial Ca^2^⁺ and nitric oxide responses to shear stress [60]. Because nitric oxide is a major regulator of vascular tone and endothelial function, defective ciliary mechanosensing may contribute to endothelial dysfunction. Primary cilia are particularly enriched in vascular regions exposed to disturbed flow, such as arterial branch points and curvatures, where atherosclerotic lesions preferentially develop [83, 84]. Loss of endothelial primary cilia promotes inflammatory endothelial activation and accelerates atherosclerosis progression [83, 85]. Recent evidence further links endothelial ciliary disruption to lipid stress. Lipid droplet accumulation in vascular endothelial cells reduces cytosolic palmitic acid availability, suppresses S-palmitoylation of ciliary proteins, and impairs ciliogenesis, thereby promoting atherosclerosis through disruption of endothelial ciliary homeostasis [85]. These findings suggest that endothelial primary cilia protect vascular homeostasis by coordinating flow sensing, inflammatory control, and lipid-dependent ciliary maintenance.

#### Angiogenesis and vascular remodeling

Endothelial primary cilia regulate angiogenesis and vascular remodeling by integrating mechanical, hypoxic, and morphogen-related cues. Rather than requiring persistent ciliogenesis, vascular adaptation may depend on dynamic ciliary assembly and disassembly. Tubulin glutamylation enables endothelial cells to respond to shear stress and hypoxia, promoting Aurora A-dependent deciliation and VEGFA/VEGFR2 expression. Loss of centrosomal protein 41 disrupts tubulin glutamylation, impairs endothelial migration, tube formation, and vascular development, suggesting that primary cilia initially act as sensory platforms, whereas subsequent disassembly supports angiogenic remodeling [86].

Ciliary signaling also contributes to tissue-specific vascular development. In the developing retina, endothelial primary cilia support shear-responsive bone morphogenetic protein signaling and nitric oxide synthase induction, thereby promoting vascular expansion and branching [87]. These studies indicate that primary cilia coordinate mechanical, hypoxic, and biochemical signals during angiogenesis and vascular remodeling.

### Reproductive system

Successful reproduction requires precise coordination of endocrine regulation, tissue remodeling, embryo implantation, and placental development, all of which are tightly linked to metabolic adaptation. Emerging evidence suggests that primary cilia function as specialized signaling platforms that integrate hormonal, inflammatory, and metabolic cues across reproductive tissues. Disruption of ciliary signaling has been associated with endometriosis-associated infertility, preeclampsia, recurrent miscarriage, and impaired reproductive endocrine regulation.

#### Pituitary development and reproductive endocrine regulation

The pituitary gland regulates reproductive function by secreting gonadotropins, including follicle-stimulating hormone and luteinizing hormone. Primary cilia are essential for pituitary development and endocrine homeostasis, largely through Hedgehog signaling. Ciliary dysfunction impairs Hedgehog signaling, leading to pituitary hypoplasia, increased apoptosis and tissue regression, and defective anterior pituitary cell specification [88, 89]. Postnatally, disruption of BBS4 or pituitary IFT88 also causes pituitary hypoplasia and altered gonadotroph composition [11]. These findings suggest that primary cilia shape reproductive endocrine function by regulating pituitary development and postnatal endocrine cell balance.

#### Decidualization failure

Decidualization is the differentiation of endometrial stromal cells into specialized decidual cells during the secretory phase and is essential for embryo implantation and pregnancy maintenance [90]. Failure of this process can impair uterine receptivity and contribute to infertility. In endometriosis, a common gynecological disorder characterized by the growth of endometrial-like tissue outside the uterus, defective decidualization is considered a major contributor to endometriosis-associated infertility, together with other abnormalities in the reproductive microenvironment [91–93]. Primary cilia contribute to decidualization by organizing stromal signaling pathways. In mice, stromal primary cilia increase in number and length during early pregnancy and mediate epithelial–stromal communication through sonic hedgehog-dependent Hedgehog signaling [94]. In human endometrial stromal cells, EP4 localizes to primary cilia and mediates prostaglandin E2-cAMP signaling to promote decidualization. In women with endometriosis, endometrial stromal cell primary cilia are shorter, and decidualization responses are reduced [95]. Mechanistically, elevated transforming growth factor beta 1 (TGF-β1) suppresses ciliogenesis by downregulating ciliogenic effector KIF3B. Loss of primary cilia disrupts ciliary cAMP-dependent expression of decidualization-associated proteins. Clinically, successful pregnancy after in vitro fertilization-embryo transfer is associated with more abundant ciliated stromal cells and longer cilia, suggesting that defective endometrial ciliogenesis may contribute to impaired uterine receptivity [95].

#### Preeclampsia

Preeclampsia is a pregnancy-specific hypertensive disorder that usually occurs after 20 weeks of gestation and is a major cause of maternal and perinatal morbidity [96]. Although its pathogenesis is heterogeneous, abnormal placentation is a central feature, particularly in early-onset disease. During normal placentation, extravillous trophoblasts invade the maternal decidua and remodel spiral arteries into low-resistance vessels that support adequate placental perfusion. In preeclampsia, impaired trophoblast invasion and incomplete spiral artery remodeling contribute to placental hypoxia, oxidative stress, and inflammatory activation [97]. In trophoblast cells, cilia-dependent endocrine gland-derived vascular endothelial growth factor signaling activates ERK1/2 and increases metalloproteinase (MMP) 2 and MMP9 expression, thereby promoting invasive capacity [98]. However, trophoblast primary cilia are shortened in preeclamptic placentas. Pro-inflammatory cytokines such as interleukin-6 and tumor necrosis factor-α reduce ciliary length, impair Hedgehog signaling, and decrease trophoblast motility and MMP2/MMP9 expression, suggesting that inflammatory disruption of ciliary signaling may contribute to defective placentation [99].

Ciliary dysfunction in preeclampsia is not limited to trophoblasts. Human chorionic villus mesenchymal stromal cells are multipotent progenitor cells that support placental vascularization and protect endothelial cells from oxidative stress [100]. Cells isolated from preeclamptic placentas exhibit reduced ciliation, shortened primary cilia, impaired Hedgehog signaling, diminished differentiation potential, and weaker pro-angiogenic activity [101]. These studies suggest that placental primary cilia coordinate trophoblast invasion, stromal cell function, and vascular development, and that disruption of these ciliary functions may contribute to the pathogenesis of preeclampsia.

#### Recurrent miscarriage

Recurrent miscarriage is defined as two or more consecutive failed clinical pregnancies documented by ultrasound or histopathology. Emerging evidence suggests that ciliary dysfunction may contribute to recurrent miscarriage by impairing maternal decidualization and trophoblast invasion. Decidual tissues from patients with spontaneous abortion also show reduced ciliation, shortened primary cilia, and impaired TGF-β signaling, while tumor necrosis factor-α can shorten cilia in human endometrial stromal cells [102, 103]. Cilia-dependent cAMP signaling is also required for decidualization, and disruption of IFT88 or CEP164 reduces decidual marker expression [104].

Genetic evidence further links defective ciliary trafficking to pregnancy loss. Deleterious variants in *DYNC2H1*, which encodes the heavy chain of cytoplasmic dynein-2, have been identified in euploid recurrent pregnancy loss [105]. *DYNC2H1* deficiency disrupts dynein-2-mediated ciliary trafficking, resulting in abnormal ciliary architecture and accumulation of intraflagellar transport and Hedgehog signaling proteins within primary cilia [106]. Trophoblast ciliogenesis is also regulated by miRNAs. Elevated miR-20b-5p suppresses autophagy-dependent ciliogenesis by targeting autophagy initiator proteins, thereby reducing trophoblast migration and invasion [107]. Similarly, miR-155-5p promotes centrosome amplification, inhibits primary cilium formation, and impairs trophoblast motility [108]. Overall, these findings suggest that recurrent miscarriage may partly result from ciliary defects that compromise decidualization, trophoblast invasion, and embryo–maternal communication.

### Metabolic disorders

Primary cilia coordinate metabolic homeostasis by integrating hormonal, nutritional, and environmental cues. Through the organization of GPCRs, neuroendocrine receptors, and nutrient-responsive signaling pathways, they regulate tissue homeostasis and inter-organ communication in metabolic organs, including the hypothalamus, adipose tissue, pancreatic islets, and kidney [13, 109]. Disruption of ciliary structure or signaling can therefore impair energy balance, glucose metabolism, and mitochondrial adaptation, contributing to obesity, type 2 diabetes, and metabolic ciliopathies.

#### Obesity

Neuronal primary cilia regulate appetite and energy expenditure by organizing hypothalamic leptin-melanocortin signaling. Melanocortin 4 receptor (MC4R) localizes to primary cilia of paraventricular hypothalamic neurons, where cAMP signaling mediates its anorexigenic effects. Disruption of neuronal cilia or ciliary MC4R signaling causes hyperphagia and obesity [110, 111]. Proper ciliary trafficking also supports MC4R activity, as melanocortin-2 receptor accessory protein 2 promotes MC4R trafficking to neuronal cilia, while G protein-coupled receptor 45 enhances ADCY3-dependent cAMP signaling and melanocortin responses [112, 113]. In addition, MC4R-containing cilia shorten with aging and overnutrition, reducing melanocortin sensitivity, energy expenditure, and thermogenesis [114]. These findings indicate that defective ciliary receptor trafficking, cAMP signaling, and ciliary structure can impair central appetite control.

Beyond the brain, primary cilia also regulate adipose progenitor function and tissue remodeling. In *Bbs8*-deficient mice, impaired ciliary Hedgehog signaling shifts adipocyte progenitors toward a fibrogenic state, disrupting white adipose tissue homeostasis and contributing to obesity [115]. Primary cilia can also negatively regulate adipogenesis through ciliary EP4 signaling, which activates Rho-associated coiled-coil-containing protein kinase 2 and suppresses the cytoskeletal remodeling required for adipocyte differentiation [116]. In human obesity, adipose-derived mesenchymal stromal/stem cells exhibit shortened and functionally impaired primary cilia, accompanied by reduced migration, differentiation, and responsiveness to cilia-dependent signals [109, 117]. Obesity-associated stressors, including hypoxia, inflammatory cytokines, and TGF-β, can further impair ciliogenesis through pathways involving Aurora kinase A and ERK1/2 [109, 117, 118]. Together, these findings suggest that obesity involves both central ciliary defects in appetite regulation and peripheral ciliary dysfunction in adipose progenitor remodeling.

#### Diabetes

Type 2 diabetes is characterized by impaired glucose homeostasis and progressive β-cell dysfunction, in which β-cells fail to compensate for increased metabolic demand. Given the role of primary cilia in regulating islet hormone secretion and paracrine communication, diabetes-associated ciliary defects may contribute to β-cell dysfunction. Several lines of evidence support this connection. Phosphorylated EPHA3 levels are elevated in islets from individuals with type 2 diabetes compared with normoglycemic donors, suggesting a potential link between dysregulated EPHA signaling, ciliary function, and islet dysfunction [44]. Human type 2 diabetic islets also display shortened β-cell primary cilia and reduced β-cell-δ-cell interactions, which may disrupt local somatostatin-SSTR3 signaling and contribute to β-cell dysfunction [43]. Given that several metabolic and paracrine GPCRs localize to β-cell primary cilia, diabetes-associated ciliary shortening may perturb compartmentalized cAMP and Ca^2^⁺ signaling required for hormone secretion [42, 43, 48]. Collectively, these findings suggest that ciliary dysfunction contributes to type 2 diabetes progression by impairing β-cell adaptation, insulin secretory regulation, and endocrine communication within the islet.

#### Metabolic ciliopathies and mitochondrial dysfunction

Classical ciliopathies provide strong evidence that ciliary dysfunction can produce systemic metabolic phenotypes. Classical ciliopathies, including Bardet-Biedl syndrome, are often associated with obesity and metabolic abnormalities, supporting a role for primary cilia in systemic energy balance [50, 119]. At the cellular level, disruption of ciliary trafficking can impair receptor localization, neuroendocrine signaling, and mitochondrial function, thereby affecting nutrient sensing and tissue-level metabolic control.

Renal ciliopathies further illustrate the connection between ciliary dysfunction, mitochondrial metabolism, and tissue pathology. In polycystic kidney disease, disruption of ciliary proteins such as PC1 and PC2 is associated with abnormal mechanosensory signaling, metabolic reprogramming, and mitochondrial dysfunction [120, 121]. Cilia-deficient renal tubule cells also show mitochondrial defects and aberrant tryptophan metabolism, suggesting that loss of ciliary signaling can prime renal epithelial cells for metabolic injury [122]. In Bardet-Biedl syndrome, BBSome dysfunction alters mitochondrial dynamics and function, providing a mechanistic link between ciliary trafficking defects and mitochondrial metabolic stress [123]. These findings suggest that metabolic dysfunction in ciliopathies may reflect not only abnormal developmental signaling but also impaired cilia-dependent control of mitochondrial homeostasis.

Ciliary EV signaling may provide an additional disease-relevant mechanism. Primary cilia can release EVs or ectosomes, and BBSome-related defects can alter vesicle release and cargo composition [65, 69, 124]. Although direct control of miRNA cargo selection by primary cilia remains incompletely established, disease-associated changes in ciliary trafficking may disrupt EV-mediated communication. This mechanism may be relevant to ciliopathies and neurological disease, where both cilia and extracellular vesicles contribute to intercellular signaling [125].

### Primary cilia in cancer chemoresistance and metastasis

In cancer, the relationship between primary cilia and metabolism is highly context-dependent. Many cancers show reduced primary cilium frequency or complete loss of primary cilia [126–129]. This reduction may reflect the inverse relationship between ciliogenesis and cell-cycle progression, because primary cilia usually assemble in quiescent cells and disassemble before cell-cycle re-entry [130–132]. However, ciliary loss in cancer is not always permanent. Under treatment or genotoxic stress, some cancer cells can reassemble or elongate primary cilia, suggesting that ciliary regrowth may act as an adaptive stress response [133]. In this context, primary cilia may provide a specialized signaling platform that allows cancer cells to sense extracellular stress and remodel survival or invasion pathways. Hypoxia-associated ciliary regulation may be particularly relevant in tumor contexts. The VHL tumor suppressor links oxygen sensing to primary cilium maintenance by regulating HIF-α degradation and supporting microtubule stability [35, 36]. Loss of pVHL can therefore affect both pseudohypoxic signaling and ciliary integrity, providing a possible explanation for the overlap between tumorigenic and cystic features in VHL-associated disease. In addition, HIF-2α can interact with IFT88 under hypoxic conditions and influence MEK/ERK signaling through a primary cilium-associated mechanism [37]. These findings suggest that tumor hypoxia and pseudohypoxic signaling may remodel primary cilia in a context-dependent manner.

#### Chemoresistance

Primary cilia have been linked to resistance against diverse anticancer therapies. Resistant lung cancer cells often display increased and elongated primary cilia, and primary cilia mediate multiple kinase inhibitor resistance mechanisms [134]. In pancreatic ductal adenocarcinoma, cisplatin-resistant cells regrow primary cilia through DNA damage-associated stress signaling, and disruption of ciliogenesis sensitizes resistant cells to cisplatin [135]. In glioblastoma, inhibition of ciliogenesis or depletion of ciliogenesis regulators such as Pericentriolar material 1 increases sensitivity to temozolomide and ionizing radiation, suggesting that primary cilia can support survival under genotoxic stress [136, 137]. In triple-negative breast cancer, epithelial-to-mesenchymal transition (EMT)-associated ciliary signaling has also been linked to stem-like states and therapeutic resistance [138]. These studies suggest that therapy-induced or stress-induced ciliogenesis can contribute to cancer cell plasticity and drug tolerance.

Cancer-associated ciliary remodeling can also be driven by post-transcriptional mechanisms. In cholangiocarcinoma, dysregulation of miR-22 and miR-433 induces Histone deacetylase 6 overexpression and ciliary loss [139]. Because histone deacetylase 6 promotes microtubule deacetylation and ciliary disassembly, this mechanism provides a link between miRNA dysregulation, ciliary loss, and cancer progression. Thus, cancer-associated ciliary remodeling can occur in different directions: some tumors lose primary cilia during proliferation, whereas others reassemble or retain cilia under stress to support survival or invasion.

#### Metastasis

Primary cilia regrowth may promote metastatic adaptation under nutrient-stressed tumor microenvironments. In pancreatic ductal adenocarcinoma, glutamine deficiency induces melanophilin, which localizes to centrosomes and promotes primary cilia regrowth. These reassembled cilia activate a PLCγ1-dependent signaling axis that enhances metastatic potential, linking amino acid deprivation, ciliary remodeling, and cancer dissemination [17]. Checkpoint kinase 2 has also been reported to coordinate autophagy activation and Aurora kinase A degradation to regulate primary cilia for cell invasion under metabolic stress [140]. Beyond pancreatic cancer, ciliated tumor cell states have been associated with EMT-like plasticity and invasive behavior. EMT-associated programs induce ciliogenesis and activate WNT signaling, which potentially facilitates the development of claudin-low breast cancers [141]. In bladder cancer, primary cilia are present in invasive tumor tissues and correlate with EMT and Hedgehog pathway activation; ciliated cells have also been observed crossing the basement membrane in non-muscle-invasive bladder cancer, suggesting a potential role for primary cilia in migration and invasiveness [142, 143]. Together, these studies support the concept that primary cilia can be re-engaged by cancer cells to coordinate metabolic stress sensing, EMT-associated plasticity, and pro-metastatic signaling.

### Nervous system: neurodegeneration and aging

In the nervous system, primary cilia are important not only during development but also for maintaining neuronal signaling, metabolic adaptation, and stress resilience. Neurons and glial cells are long-lived and highly specialized cells that require tight coordination between extracellular cues, intracellular signaling, mitochondrial quality control, and proteostasis. Primary cilia may contribute to these processes by organizing neuromodulatory receptors, second-messenger pathways, and stress-responsive signaling modules.

#### Primary cilia in neurodevelopment and neuronal maturation

During nervous system development, primary cilia act as stage-specific signaling hubs that coordinate neural progenitor proliferation, neuronal differentiation, migration, polarity, and circuit maturation. At the progenitor stage, cilia-mediated sonic hedgehog and GLI signaling regulate neural progenitor proliferation and cortical size [144]. As neural progenitors differentiate, primary cilia integrate Wnt/β-catenin, cAMP, and GPCR-related signaling pathways to support neuronal differentiation, migration, and polarity [145, 146]. During neuronal maturation, structurally intact primary cilia enriched with adenylate cyclase 3 are required for dendritic arborization of developing neocortical neurons, suggesting that ciliary cAMP signaling contributes to neuronal morphology and circuit formation [147]. Beyond embryonic development, primary cilia continue to regulate synaptic integration of adult-born neurons [148]. Accordingly, ciliary dysfunction can produce neurological ciliopathies such as Joubert syndrome, Meckel-Gruber syndrome, and Bardet-Biedl syndrome, in which mutations affecting ciliary structure, trafficking, or signaling cause brain malformations, cognitive impairment, and neurodevelopmental abnormalities [149].

#### Primary cilia maintain neuronal signaling and metabolic homeostasis

Beyond development, primary cilia remain functionally important in mature neurons by acting as sensory and signaling compartments that detect neurochemical, hormonal, and metabolic cues. Mature neurons are highly polarized and long-lived; therefore, they require mechanisms that can integrate extracellular signals with intracellular metabolic and stress-adaptive responses. Primary cilia provide such a platform by concentrating specific receptors and downstream signaling molecules within a specialized membrane compartment [146]. Neuronal cilia are enriched with neuromodulatory receptors, making them sensitive to neurotransmitters and neuropeptides, including dopamine, serotonin, and somatostatin [150–152]. This compartmentalization allows cilia to convert extracellular inputs into localized second-messenger responses, including cAMP, Ca^2^⁺-associated signaling, and Hedgehog/GPCR-dependent pathways. Through these pathways, neuronal primary cilia may influence neuronal excitability, synaptic function, neuroendocrine responses, and metabolic adaptation. Thus, beyond their developmental roles, primary cilia serve as signaling organelles that couple environmental sensing to neuronal homeostasis in the mature nervous system.

#### Ciliary dysfunction in neurodegenerative diseases

When cilia-dependent homeostatic signaling is disrupted, neurons and glial cells may lose the ability to adapt to metabolic, oxidative, and proteostatic stress, thereby increasing vulnerability to neurodegenerative disease. Alzheimer’s disease provides one emerging example. In Alzheimer’s disease mouse models, neuronal primary cilia near Aβ plaques are shortened, and microglial ciliary signaling has been implicated in secretory regulation and extracellular proteostasis [153, 154]. These findings suggest that Alzheimer’s disease pathology may perturb ciliary morphology and signaling in a cell type- and disease stage-dependent manner.

In Parkinson’s disease, primary cilia have been linked to dopaminergic neuron survival and stress adaptation. Parkinson’s disease-associated leucine-rich repeat kinase 2 inhibits primary cilium formation and impairs cilia-dependent sonic hedgehog signaling [155]. Primary cilia also mediate cellular stress responses and autophagy to promote dopaminergic neuron survival, whereas the loss of primary cilia impairs the clearance of damaged mitochondria and exacerbates neuronal degeneration [156]. In addition, mitochondrial DNA depletion in astrocytes activates a mitochondrial integrated stress response and induces ciliogenic transcriptional programs, supporting a bidirectional relationship between mitochondrial stress and ciliary remodeling in glial cells [157]. These studies suggest that ciliary dysfunction may compromise neuronal and glial stress resilience by weakening sonic hedgehog signaling, mitochondrial quality control, and adaptive remodeling.

Compared with Alzheimer’s and Parkinson’s disease, the role of primary cilia in amyotrophic lateral sclerosis remains less well defined. However, recent studies link amyotrophic lateral sclerosis-associated genes to dysregulated ciliogenesis. Variants in cilia- and flagella-associated protein 410 (CFAP410, formerly C21ORF2) reduce cilia number and length in motor neurons, impairing sonic hedgehog signaling and neuromuscular junction formation [158]. Similarly, mutations in NIMA-related kinase 1 cause ciliary abnormalities, reduced tubulin acetylation, and aberrant cell-cycle re-entry in patient-derived motor neurons [159]. These findings suggest that impaired ciliogenesis and ciliary signaling may contribute to motor neuron dysfunction in amyotrophic lateral sclerosis.

Overall, primary cilia may influence neuronal vulnerability not only through developmental mechanisms but also by regulating metabolic adaptation, intracellular trafficking, mitochondrial quality control, and stress resilience during aging and neurodegeneration.

Taken together, current evidence for the involvement of primary cilia across these biological and disease contexts ranges from established genetic causation and direct experimental mechanisms to emerging disease-associated observations. Supplementary Table S1 provides an evidence-stratified summary of the biological context, mechanistic relationship, experimental system, and level of supporting evidence.

## Future perspectives: from environmental sensing to metabolic intervention

Despite growing recognition of primary cilia as sensory organelles and metabolic signaling hubs, several fundamental questions remain unresolved. Addressing these questions will require not only improved experimental tools but also a clearer conceptual framework for understanding how ciliary signals are measured, integrated, and therapeutically manipulated.

A major limitation in the current field is that many mechanistic insights are derived from static imaging, endpoint analyses, or simplified in vitro systems. However, primary cilia are highly dynamic organelles whose signaling output is shaped by temporal and spatial changes in the tissue microenvironment. Future studies should enable real-time visualization of ciliary signaling events in vivo, including localized changes in Ca^2^⁺, cAMP, receptor activity, and metabolic flux [15, 160, 161]. In parallel, tissue-specific and temporally controlled cilia manipulation models will be essential to distinguish developmental requirements from acute cilia-mediated adaptation [13, 75]. Integration of ciliary perturbation with metabolomics, spatial omics, and single-cell multi-omics may further reveal how distinct ciliary states shape cellular metabolism across heterogeneous tissues [15, 162].

Another important challenge is to understand how different environmental cues are integrated within the ciliary compartment. Nutrient stress, mechanical stimulation, and oxygen availability have each been shown to regulate ciliary structure or signaling, but it remains unclear whether these inputs act through independent signaling modules or converge on shared molecular nodes [16, 25, 37]. Future work should determine how ciliary channels, GPCRs, trafficking machinery, lipid composition, metabolic sensors, and structural remodeling collectively encode environmental information into distinct signaling outputs [5, 163, 164]. Defining these integration mechanisms will be critical for understanding how primary cilia translate multiple extracellular inputs into coordinated metabolic decisions.

A major translational opportunity is to exploit ciliary signaling to reshape pathological metabolic states. Rather than targeting individual downstream pathways, modulation of ciliogenesis, ciliary trafficking, GPCR localization, Ca^2^⁺ signaling, autophagy, or cilia-associated metabolic pathways may allow broader rewiring of cellular adaptation programs [5, 135, 137, 163, 164]. Such strategies may be relevant to metabolic disorders, cancer, and neurodegenerative diseases, in which ciliary remodeling can influence stress responses and disease progression. However, the consequences of manipulating these processes are highly dependent on cell type, tissue context, and disease stage, and ciliary signaling may exert protective or pathogenic effects in different settings. Broad stimulation or inhibition of ciliary activity could therefore disrupt homeostasis in tissues where cilia perform protective functions. Future strategies should prioritize cell- and tissue-specific delivery, pathway-selective targeting, and temporally controlled modulation guided by disease stage and appropriate biomarkers. Defining when ciliary remodeling is adaptive or maladaptive will be essential for developing safe and effective cilia-directed therapies.

## Conclusion

In conclusion, primary cilia should be viewed not only as sensory antennae but also as dynamic metabolic signaling hubs that couple environmental information to cellular adaptation. By adjusting their assembly, length, molecular composition, and compartmentalized signaling output in response to nutrient availability, mechanical forces, and oxygen tension, primary cilia coordinate diverse metabolic processes, including glucose and lipid homeostasis, Ca^2^⁺ and second-messenger signaling, miRNA-associated regulation, mitochondrial quality control, and stress adaptation. When this sensory-metabolic axis is disrupted, ciliary signaling can contribute to cardiovascular, reproductive, metabolic, oncogenic, and neurodegenerative disease processes. However, ciliary remodeling is not uniformly beneficial or harmful; its consequences depend on cell type, tissue context, environmental stress, and disease stage. Defining when primary cilia preserve homeostasis and when they promote maladaptive remodeling will therefore be central to translating cilia biology into therapeutic strategies. With advances in live ciliary signaling reporters, tissue-specific models, and spatial multi-omics, future studies are poised to move the field from describing ciliary structure toward decoding and manipulating ciliary signaling states in health and disease.

## Supplementary Information

Supplementary Material 1.

## Data Availability

No datasets were generated or analysed during the current study.

## Abbreviations

Aβ: Amyloid beta
ACC1: Acetyl-CoA carboxylase 1
ADCY3: Adenylyl cyclase 3
AMPK: AMP-activated protein kinase
ATP: Adenosine triphosphate
BBS: Bardet–Biedl syndrome
BDNF: Brain-derived neurotrophic factor
cAMP: Cyclic adenosine monophosphate
CEP: Centrosomal protein
CFAP410: Cilia- and flagella-associated protein 410
CP110: Centriolar coiled-coil protein 110
DRP1: Dynamin-related protein 1
EMT: Epithelial-to-mesenchymal transition
EP4: Prostaglandin E receptor 4
EPAC: Exchange protein directly activated by cAMP
EphA: Ephrin type-A
EV: Extracellular vesicle
FFAR4: Free fatty acid receptor 4
GLP-1R: Glucagon-like peptide-1 receptor
GPCR: G protein-coupled receptor
HIF: Hypoxia-inducible factor
IFT: Intraflagellar transport
IFT88: Intraflagellar transport protein 88
IP_3_: Inositol 1,4,5-trisphosphate
KIF3A/KIF3B: Kinesin family members 3A and 3B
LKB1: Liver kinase B1
MC4R: Melanocortin 4 receptor
MMP: Matrix metalloproteinase
mTORC1: Mechanistic target of rapamycin complex 1
mtDNA: Mitochondrial DNA
NEK4: NIMA-related kinase 4
NPHP: Nephronophthisis
OXPHOS: Oxidative phosphorylation
PC1/PC2: Polycystin-1 and polycystin-2
PI3K: Phosphoinositide 3-kinase
PKA: Protein kinase A
PLC: Phospholipase C
pVHL: Von Hippel–Lindau protein
SMO: Smoothened
SREBP1: Sterol regulatory element-binding protein 1
SSTR3: Somatostatin receptor 3
TFAM: Mitochondrial transcription factor A
TFEB: Transcription factor EB
TGF-β1: Transforming growth factor beta 1
TRP: Transient receptor potential
TRPC3: Transient receptor potential canonical 3
TRPV4: Transient receptor potential vanilloid 4
TULP3: Tubby-like protein 3
VEGF-A: Vascular endothelial growth factor A
VEGFR2: Vascular endothelial growth factor receptor 2
