# Supplementary material for: Primary cilium as a sensory organelle and metabolic hub in health and diseases
Source: J Biomed Sci. 2026 Oct 7;33:92. doi: 10.1186/s12929-026-01291-6 (PMC13644013; doi:10.1186/s12929-026-01291-6)
Supplement: Supplementary file 1 [file 12929_2026_1291_MOESM1_ESM.docx]

**Supplementary Table S1. Evidence-stratified summary of primary cilia–disease relationships across organ systems.** Primary cilia–disease relationships are grouped according to the type and strength of supporting evidence.

| 1. **Genetic ciliopathies or cilia-first disorders** | | | | |
| --- | --- | --- | --- | --- |
| **Disease or biological context** | **Evidence layer** | **Mechanistic relationship** | **Experimental system** | **References** |
| Congenital laterality and multiorgan developmental defects | Genetic | Pathogenic variants in *NPHP3*, *NPHP4*, and *INVS/NPHP2* were associated with laterality abnormalities, congenital heart malformations, nephronophthisis, and multiorgan dysplasia, genetically linking NPHP-associated ciliary dysfunction to congenital developmental defects. | Human genetic cohorts and pedigrees; selected functional studies | Bergmann et al. (2008); French et al. (2012); Otto et al. (2003) |
| Congenital cardiac and laterality defects | Mechanistic | Mutations affecting primary cilia disrupted left-right patterning or cilia-dependent Hedgehog signaling and caused atrioventricular septal defects through distinct developmental mechanisms. | Mouse models | Burnicka-Turek et al. (2016) |
| Bardet-Biedl syndrome and receptor trafficking | Mechanistic | Loss of BBS proteins prevented selected GPCRs from entering primary cilia, establishing BBSome-dependent receptor trafficking as a determinant of neuronal ciliary signaling. | Mouse brain tissue and cultured cells | Berbari et al. (2008) |
| Adipocyte progenitor differentiation and adipose remodeling | Mechanistic | *Bbs8* deficiency dysregulated ciliary Hedgehog signaling and promoted a fibrogenic adipocyte-progenitor fate, whereas ciliary EP4 activation suppressed adipogenesis, demonstrating context-dependent ciliary control of adipose progenitor differentiation. | Mouse adipocyte progenitors, 3T3-L1 cells, and mouse models | Sieckmann et al. (2025); Lee and Hilgendorf (2025) |
| Bardet-Biedl syndrome and mitochondrial dysfunction | Mechanistic and correlative | BBSome deficiency caused mitochondrial hyperfusion and metabolic dysfunction in patient fibroblasts and experimental models, supporting a functional link between BBSome disruption and mitochondrial dynamics. | Patient fibroblasts, mouse neuronal cells, and mouse models | Guo et al. (2023) |
| BBS-associated extracellular-vesicle communication | Mechanistic | *Bbs4* or *Bbs6* loss increased extracellular-vesicle release and altered vesicular cargo; similar release changes occurred in BBS10 patient-derived renal cells, although selective miRNA secretion by cilia was not established. | Mouse kidney cells and BBS10 patient-derived renal epithelial cells | Volz et al. (2021) |
| **2. Strong mechanistic preclinical evidence** | | | | |
| **Disease or biological context** | **Evidence layer** | **Mechanistic relationship** | **Experimental system** | **References** |
| Cardiac development and chamber maturation | Mechanistic | *Ift88*/*Ift20* depletion impaired ciliogenesis, Hedgehog signaling, and cardiomyocyte differentiation, whereas disruption of endocardial ciliary flow sensing reduced Notch signaling and ventricular trabeculation. | Mouse cardiomyocyte differentiation and zebrafish models | Clement et al. (2009); Samsa et al. (2015) |
| Pituitary development | Mechanistic | *Bbs4* loss or pituitary *Ift88* disruption impaired postnatal ciliogenesis, pituitary growth, and endocrine-cell composition. | Mouse models | Brewer et al. (2026) |
| Endothelial mechanosensing and vascular development | Mechanistic | Disruption of ciliary polycystin-2, CEP41-dependent tubulin glutamylation, or β-arrestin/BMPR2 signaling impaired shear-responsive Ca²⁺/nitric-oxide signaling, endothelial migration, angiogenesis, or vascular development. | Endothelial cells and mouse or zebrafish models | AbouAlaiwi et al. (2009); Ki et al. (2020); Park et al. (2022) |
| Atherosclerosis | Mechanistic | Endothelial *Ift88* deletion enhanced inflammatory activation and atherosclerosis, while lipid-droplet-mediated palmitic-acid sequestration reduced ciliary-protein S-palmitoylation and ciliogenesis, identifying complementary cilia-to-disease and metabolic-stress-to-cilia mechanisms. | Mouse atherosclerosis models and endothelial cells | Dinsmore and Reiter (2016); Tan et al. (2024) |
| Embryonic patterning and development | Mechanistic | *Fuz* or *Dyrk2* deficiency impaired ciliogenesis and Hedgehog signaling, producing defective embryonic tissue patterning and multiorgan developmental abnormalities. | Mouse genetic models | Gray et al. (2009); Yoshida et al. (2020) |
| Uterine receptivity, decidualization, and pregnancy loss | Mechanistic and correlative | Disruption of stromal *IFT88* or *CEP164* impaired cilia-dependent Hedgehog, PGE2-cAMP, TGF-β, and progesterone-receptor signaling and reduced decidualization; spontaneous-abortion decidua showed corresponding ciliary and signaling defects. | Human uterine tissues and cells, and mouse models | Li et al. (2023); Yu et al. (2024); Lee et al. (2025) |
| Endometriosis-associated infertility | Mechanistic and correlative | TGF-β1 exposure or *IFT88*/*CEP164* disruption shortened or removed stromal-cell cilia and impaired ciliary EP4-cAMP decidual signaling; endometriosis and unsuccessful IVF outcomes were associated with reduced ciliation. | Human tissues and primary cells, and mouse models | Hou et al. (2026) |
| Preeclampsia and placental dysfunction | Mechanistic and correlative | Preeclamptic placental trophoblasts and stromal cells had fewer or shorter cilia and impaired Hedgehog-related migration, invasion, differentiation, or angiogenic support. Inflammatory exposure and *IFT88* depletion reproduced selected defects, but a causal role in disease progression remains unresolved. | Human placental tissues, primary cells, and trophoblast models | Ritter et al. (2020); Romberg et al. (2022) |
| Central regulation of obesity | Mechanistic | Neuronal-cilium loss, impaired MC4R trafficking, GPR45-Gαs-ADCY3 perturbation, or aging/overnutrition-induced shortening of MC4R-positive cilia reduced melanocortin signaling and altered appetite, energy expenditure, thermogenesis, and body weight. | Mouse models and neuronal cell systems | Wang et al. (2021); Bernard et al. (2023); Xun et al. (2025); Oya et al. (2024) |
| Obesity-associated adipose stromal dysfunction | Mechanistic and correlative | Adipose stromal cells from individuals with obesity exhibited shortened or dysfunctional cilia, reduced ADCY3, and impaired motility or differentiation; ADCY3 activation or inhibition of aurora kinase A/ERK partially restored ciliary and cellular phenotypes. | Human primary adipose-derived stromal cells | Ritter et al. (2018); Kreis et al. (2025); Ritter et al. (2019) |
| Type 2 diabetes and islet dysfunction | Mechanistic and correlative | β-cell-specific *Ift88* deletion disrupted cilia-dependent endosomal EphA processing and glucose-stimulated insulin secretion, while human diabetic islets showed increased phosphorylated EPHA3 as an associative counterpart. | Mouse β-cell models and human primary islets | Volta et al. (2019) |
| Renal metabolic dysfunction | Mechanistic | *Ift88* or *Exoc5* disruption removed renal tubular cilia, impaired mitochondrial respiration and tryptophan metabolism, and increased susceptibility to ischemic injury. | Mouse models and renal epithelial cells | Zuo et al. (2024) |
| Cancer stress adaptation and treatment resistance | Mechanistic and correlative | Genotoxic stress, kinase inhibition, cisplatin, PCM1-dependent ciliogenesis, or EMT altered primary cilia in resistant cancer models; disrupting ciliogenesis or ciliary signaling increased sensitivity to chemotherapy, radiation, or targeted inhibitors in selected contexts. | Human cancer cell lines, patient-derived cells or organoids, tumor biopsies, and functional models | Chen et al. (2021); Jenks et al. (2018); Chao et al. (2022); Hoang-Minh et al. (2016); Wei et al. (2022); Tessier et al. (2025) |
| Pancreatic ductal adenocarcinoma progression | Mechanistic | HDAC2 inhibition restored cilia through reduced aurora kinase A and suppressed proliferative behavior, whereas glutamine deprivation induced melanophilin-dependent ciliary regrowth that promoted invasion and metastasis, demonstrating context-dependent effects of ciliary remodeling. | Human pancreatic cancer cells and mouse models | Kobayashi et al. (2017); Chao et al. (2025) |
| Esophageal squamous cell carcinoma | Mechanistic and correlative | PRDX1 was elevated in tumors and activated a ciliary-disassembly pathway; PRDX1 perturbation altered ciliation and cancer-cell behavior. | Human tumor tissues and esophageal cancer cells | Chen et al. (2020) |
| miRNA- and EMT-associated ciliary remodeling in cancer | Mechanistic and correlative | Loss of miR-22/miR-433 promoted HDAC6-dependent ciliary loss in cholangiocarcinoma, whereas epithelial-mesenchymal transition induced GLIS2-dependent ciliogenesis in claudin-low breast tumorigenesis; both altered tumor-related signaling and behavior. | Human tumors and cancer cells, mouse mammary models, and human datasets | Mansini et al. (2018); Wilson et al. (2021) |
| Neuronal development and structural maturation | Mechanistic | Loss of neuronal cilia or ciliary GLI3, ADCY3/cAMP, or Akt-CREB signaling disrupted cortical growth, dendritic arborization, synaptic integration of adult-born neurons, or axon-initial-segment organization. | Mouse models, primary neurons, and ex vivo brain preparations | Wilson et al. (2012); Guadiana et al. (2013); Kumamoto et al. (2012); Wang et al. (2025) |
| Neuronal receptor signaling, synaptic function, and behavior | Mechanistic | Manipulating ciliary neuropeptide-receptor signaling altered excitatory synaptic strength, whereas loss of dopamine D1 receptor localization to cilia disrupted signaling and locomotor responses. | Mouse models, cortical or striatal neurons, and ex vivo brain preparations | Tereshko et al. (2021); Stubbs et al. (2022) |
| Parkinson disease | Mechanistic and correlative | Pathogenic LRRK2 reduced ciliogenesis and Sonic Hedgehog responsiveness, including in patient-derived cells; separately, mitochondrial stress induced autophagy-dependent ciliary elongation that protected dopaminergic neurons in mouse models. | Patient-derived and experimental cells, and mouse models | Dhekne et al. (2018); Bae et al. (2019) |
| Alzheimer disease | Mechanistic and correlative | Neuronal cilia were shortened near amyloid-β plaques and SSTR3 deletion worsened selected pathology, while microglial *Ift88* disruption altered extracellular-vesicle, lysosomal, and secretory responses to amyloid-β. | Mouse Alzheimer models, neuronal cells, and microglial models | Guo et al. (2025); Yeo et al. (2023) |
| Amyotrophic lateral sclerosis | Genetic, mechanistic, and correlative | ALS-associated *C21ORF2/CFAP410* or *NEK1* variants were linked to abnormal cilia in patient-derived cells; perturbation and rescue connected these variants to Hedgehog, neuromuscular-junction, cell-cycle, or DNA-damage phenotypes. | Human genetic cohorts, patient-derived motor neurons or fibroblasts, and functional models | De Decker et al. (2025); Noh et al. (2025) |
| **3. Emerging or predominantly disease-associated ciliary alterations** | | | | |
| **Disease or biological context** | **Evidence layer** | **Mechanistic relationship** | **Experimental system** | **References** |
| Atherosclerosis | Correlative | Endothelial primary cilia were enriched at disturbed-flow and lesion-prone sites, establishing a spatial association without independently demonstrating causation. | Mouse models | Van der Heiden et al. (2008) |
| miRNA-associated trophoblast dysfunction and miscarriage | Mechanistic and correlative | Elevated miR-20b-5p in recurrent miscarriage suppressed autophagy and ciliogenesis, while miR-155-5p promoted centrosome amplification and inhibited ciliation; both perturbations reduced trophoblast migration and invasion. | Human placental tissues and trophoblast cell models | Lin et al. (2024); Tsai et al. (2024) |
| Pregnancy-loss-associated ciliary alterations | Correlative and candidate genetic | Recurrent-miscarriage decidua showed fewer ciliated stromal cells, and recurrent early pregnancy loss was associated with candidate *DYNC2H1* variants; neither causal direction nor variant pathogenicity was established. | Human decidual tissues and genetic cohorts | Hassan et al. (2022); Qiao et al. (2016) |
| Cancer-associated ciliary loss | Correlative | Primary cilia were reduced early in breast-cancer development and largely absent from pancreatic neoplastic lesions, establishing disease-associated ciliary loss without showing that it initiates tumorigenesis. | Human breast and pancreatic tissues, and cancer models | Menzl et al. (2014); Seeley et al. (2009) |
| VHL-associated ciliary remodeling | Mechanistic | VHL loss impaired microtubule orientation and primary-cilium formation or maintenance, while VHL restoration rescued ciliogenesis; these studies did not independently establish effects on tumor progression. | Human renal epithelial cell models | Schermer et al. (2006); Lutz and Burk (2006) |
| Hypoxia-associated ciliary signaling | Mechanistic | Hypoxic HIF-2α signaling interacted with IFT88, promoted ciliary elongation, and altered MEK/ERK signaling, although the study was not conducted in a tumor model. | Mouse neuronal cells | Leu et al. (2023) |
| Bladder cancer invasion | Correlative | Primary cilia co-occurred with Hedgehog and epithelial-mesenchymal-transition markers in invasive tumor regions, supporting a spatial association rather than causal involvement. | Human bladder-tumor tissues | Iruzubieta et al. (2021) |
| Astrocytic mitochondrial stress | Mechanistic | Astrocyte-specific mitochondrial-DNA depletion activated FOXJ1/RFX ciliogenic programs and produced elongated or distorted primary cilia, demonstrating mitochondrial-stress-induced ciliary remodeling. | Mouse models and primary mouse astrocytes | Ignatenko et al. (2023) |

**Note:** Studies were classified according to the type and strength of evidence supporting the cilia–disease relationship. **(1)** **Genetic ciliopathies or cilia-first disorders** include conditions caused by pathogenic variants in established cilia-related genes or models in which ciliary dysfunction is considered a primary driver of disease. **(2) Strong mechanistic preclinical evidence** includes studies in which direct manipulation of ciliary genes, structures, or signaling pathways alters or rescues disease-relevant phenotypes in experimental models. **(3)** **Emerging or predominantly disease-associated ciliary alterations** include studies showing ciliary changes in patient samples or disease models for which the directionality or causal contribution of ciliary dysfunction remains incompletely established. Closely related studies sharing a biological context and evidence layer were consolidated within single entries; distinct evidence strengths or causal directions were retained as separate entries.
